# Transthyretin Inhibits Primary and Secondary Nucleations of Amyloid-β Peptide Aggregation and Reduces the Toxicity of Its Oligomers

**DOI:** 10.1021/acs.biomac.9b01475

**Published:** 2020-02-03

**Authors:** Seyyed Abolghasem Ghadami, Sean Chia, Francesco Simone Ruggeri, Georg Meisl, Francesco Bemporad, Johnny Habchi, Roberta Cascella, Christopher M. Dobson, Michele Vendruscolo, Tuomas P. J. Knowles, Fabrizio Chiti

**Affiliations:** † Department of Experimental and Clinical Biomedical Sciences “Mario Serio”, Section of Biochemistry, University of Florence, 50134 Florence, Italy; ‡ Department of Chemistry, Centre for Misfolding Diseases, University of Cambridge, Cambridge CB2 1EW, U.K.; § Department of Physics, Cavendish Laboratory, 19 J. J. Thomson Avenue, Cambridge CB3 0HE, U.K.

## Abstract

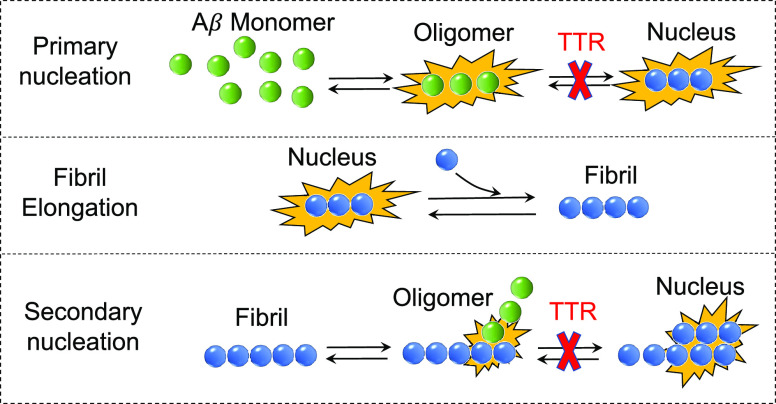

Alzheimer’s disease is associated with the deposition of the amyloid-β peptide (Aβ) into extracellular senile plaques in the brain. In vitro and in vivo observations have indicated that transthyretin (TTR) acts as an Aβ scavenger in the brain, but the mechanism has not been fully resolved. We have monitored the aggregation process of Aβ_40_ by thioflavin T fluorescence, in the presence or absence of different concentrations of preformed seed aggregates of Aβ_40_, of wild-type tetrameric TTR (WT-TTR), and of a variant engineered to be stable as a monomer (M-TTR). Both WT-TTR and M-TTR were found to inhibit specific steps of the process of Aβ_40_ fibril formation, which are primary and secondary nucleations, without affecting the elongation of the resulting fibrils. Moreover, the analysis shows that both WT-TTR and M-TTR bind to Aβ_40_ oligomers formed in the aggregation reaction and inhibit their conversion into the shortest fibrils able to elongate. Using biophysical methods, TTR was found to change some aspects of its overall structure following such interactions with Aβ_40_ oligomers, as well as with oligomers of Aβ_42_, while maintaining its overall topology. Hence, it is likely that the predominant mechanism by which TTR exerts its protective role lies in the binding of TTR to the Aβ oligomers and in inhibiting primary and secondary nucleation processes, which limits both the toxicity of Aβ oligomers and the ability of the fibrils to proliferate.

## Introduction

Alzheimer’s disease (AD) is an increasingly prevalent form of dementia that involves a loss of memory, cognitive ability, and behavioral stability. It has long been known that its main histopathological hallmarks are the presence of extracellular lesions, called senile plaques, and of intracellular bundles, called neurofibrillary tangles,^1,2^ which are known to be composed primarily of amyloid fibrils of the amyloid β (Aβ) peptide and of abnormal straight filaments or paired helical filaments formed by the protein tau, respectively.^3^

The involvement of transthyretin (TTR) in the process of Aβ fibril formation was first reported more than 20 years ago, when it was found that TTR, among the proteins present in the cerebrospinal fluid (CSF), was able to bind to Aβ_40_ and form stable complexes.^4^ Such an interaction was found to be able to inhibit amyloid fibril formation by Aβ-derived peptides in vitro, as observed with thioflavin T (ThT) fluorescence, Congo red birefringence, and transmission electron microscopy.^4^ The interaction of TTR with Aβ has been confirmed by many other studies in vitro, and it is now well established that it occurs with monomeric, oligomeric, and fibrillar forms of Aβ_40_ and Aβ_42_, with a higher affinity for oligomeric and fibrillar forms relative to monomers in solution state.^5−9^ Similarly, the inhibition of Aβ aggregation by TTR, resulting from such an interaction, has also been confirmed by many other studies in vitro,^6−8,10^ with consequent suppression of the toxic effects of Aβ.^6,8,9,11^ In addition to inhibiting Aβ fibril formation, TTR has also been shown to bind to preformed Aβ_40_ and Aβ_42_ oligomers and preformed Aβ_40_ fibrils and reduce their toxicity when added to the extracellular medium of murine primary neurons and of human neuroblastoma SH-SY5Y cells, or when injected into the hippocampi of mice, indicating a multiplicity of mechanisms through which TTR can be beneficial against the adverse effects of Aβ.^11−13^ Observations of the ability of TTR to inhibit aggregation and toxicity of Aβ have also been obtained in animal models, such as *Caenorhabditis elegans* transgenic for human Aβ_42_ and TTR^14^ and mice transgenic for mutant forms of the amyloid β precursor protein (APP) and having different levels of endogenous TTR or human TTR.^15^

The involvement of TTR in human AD is suggested by the concomitant decrease of both Aβ_42_ and TTR levels in the CSF of AD patients^16−18^ and of their accumulation and colocalization in similar areas of the cortex and hippocampus in both human AD patients and transgenic mice.^11,19^ Moreover, the expression of TTR in neurons is responsive to the expression of Aβ in both adult transgenic mice and cultured primary neurons from such mice, well before plaque formation or signs of neurodegeneration are detectable to any significant extent.^11,20^ The concentration of TTR in the CSF was also found to increase with aging in humans in the absence of dementia,^16^ suggesting that controlled expression of this protein is a response to the increasing age-dependent failure of protein homeostasis. Indeed, the recent observation that TTR expression can be induced in human neuroblastoma SH-SY5Y cells, primary hippocampal neurons from APP23 mice, and adult APP23 hippocampi (but not mouse liver or cultured hepatoma cells), following the activation of the heat shock response, is supportive of the hypothesis that TTR plays a key role in maintaining neuronal protein homeostasis and hence in protecting against neurodegeneration.^21^

Despite intensive research, the exact mechanism by which TTR modulates the behavior of Aβ and, in particular, affects the microscopic steps in the complex process of conversion of monomeric Aβ into amyloid fibrils is still not known in detail. It is also unclear if the tetrameric and monomeric forms of TTR undergo conformational changes when binding to Aβ. In this work, we have investigated the unseeded and seeded formations of Aβ_40_ fibrils in the presence of a variety of concentrations of both WT-TTR and M-TTR to establish which microscopic steps of the process of fibril formation are affected by TTR, and have also determined the conformational changes occurring in TTR during these events, as well as those associated with its interaction with preformed toxic oligomers of Aβ_42_.

## Materials and Methods

### Expression, Purification, and Site-Directed Mutagenesis of TTRs

The pMMHa plasmid containing the WT-TTR or M-TTR gene was transformed into competent BL21 DE3 Epicurian Gold cells (Agilent Technologies, Santa Clara, CA). The M-TTR gene was previously obtained by introducing two mutations at the dimer–dimer interface (F87M and L110M) in the Hu-TTR plasmid DNA by site-directed mutagenesis.^22^ Moreover, a mutant protein named W79F-M-TTR was produced by site-directed mutagenesis starting from the DNA plasmid of M-TTR using the QuickChange site-directed mutagenesis kit (Agilent Technologies, Santa Clara, CA). All DNA sequences were checked with DNA sequencing.

All TTR variants were isolated and purified following previously described procedures.^23^ In brief, the initial culture of *Escherichia coli* cells containing the plasmid was grown until cell growth was visible before inoculating 15 mL of the culture into 1.5 L of LB media with 100 μg mL^–1^ of ampicillin in 2.8 L Fernbach flasks. The cells were grown at 37 °C with vigorous shaking until OD_600_ = 1.0–1.2; they were then induced with 1 mM isopropyl β-d-thiogalactoside (IPTG) overnight at 37 °C with vigorous shaking. They were then harvested by centrifugation at 21 000*g* for 10 min at 4 °C and resuspended in 100 mL L^–1^ of culture of TBS (20 mM Tris, 0.5 M NaCl, pH 7.5 1 mM phenylmethylsulfonyl fluoride (PMSF) and 1 mM ethylenediaminetetraacetic acid (EDTA)), and then sonicated in a cold room or ice bath (3 cycles, 3 min each, with 3 s pulses, 100 amplitude, 1 min resting between cycles). Pellet cell debris was collected by centrifugation at 12 000*g* for 15 min at 4 °C, and the resuspended ammonium sulfate pellet was desalted by dialysis against 2 L of 25 mM Tris, pH 8.0, at 4 °C for 24 h using membranes with a 4 kDa molecular weight cutoff (MWCO). The resulting solutions were chromatographed on an ionic exchange, HR-Q column (23 mL) using buffer A (25 mM Tris, 1 mM EDTA, pH 8.0) and buffer B (25 mM Tris, 1 M NaCl, 1 mM EDTA, pH 8.0) with the gradient starting from 0% buffer B, changing to 20% buffer B in 1 CV, going to 35% buffer B in 9 CV, keeping 35% buffer B for 1/2 CV, and then going to 100% buffer B in 2 CV. TTR samples were collected from 21% buffer B to 35% buffer B and concentrated down to ∼20 mL using an ultrafilter and a 10 kDa MWCO membrane and were further purified by gel filtration by employing a Superdex 75 gel filtration column (GE Healthcare, Chicago, IL) at a flow rate of 1.8 mL min^–1^ and eluted in 10 mM phosphate buffer, 100 mM KCl, 1 mM EDTA, pH 7.6, 4 °C. The center of the absorbance peak was collected. The purification yield was usually ∼10–30 mg L^–1^ of LB culture. Purified proteins were stored at −20 °C in 20 mM phosphate buffer, pH 7.4. Purified proteins were checked with matrix-assisted laser desorption ionization (MALDI) mass spectrometry, and their purity was found by sodium dodecyl sulfate-polyacrylamide gel electrophoresis (SDS-PAGE) to be >95% in all cases. Protein concentrations were determined spectrophotometrically using ε_280_ = 18 450 M^–1^ cm^–1^ for M-TTR, ε_280_ = 77 600 M^–1^ cm^–1^ for WT-TTR, and ε_280_ = 12 950 M^–1^ cm^–1^ for W79F-M-TTR.

### Expression and Purification of Aβ_40_ and Aβ_42_

Aβ_40_ was expressed in the *E. coli* BL21 Gold (DE3) strain (Agilent Technologies, Santa Clara, CA) and purified as described previously^24^ with slight modifications. Briefly, the purification procedure involved sonication of *E. coli* cells, dissolution of inclusion bodies in 8 M urea, and ion exchange in batch mode on diethylaminoethyl cellulose resin followed by lyophilization. The lyophilized powder was dissolved, further purified using a Superdex 75 HR 26/60 column (GE Healthcare, Buckinghamshire, U.K.), and the fractions collected were analyzed using SDS-PAGE for the presence of the desired peptide product. The fractions with the peptide were combined, frozen using liquid nitrogen, and lyophilized again. The Aβ_40_ concentration was determined spectrophotometrically using ε_280_ = 1490 M^–1^ cm^–1^.

Aβ_42_ was purchased from Abcam (Cambridge, U.K.). The lyophilized powder was dissolved in hexafluoroisopropanol (HFIP), which was then evaporated with a nitrogen flow; the powder was then dissolved in dimethyl sulfoxide (DMSO) and reached final volume by the addition of the desired buffer. Finally, the protein was incubated at 4 °C for 24 h. Protein concentration was determined using ε_280_ = 1490 mol^–1^ cm^–1^. Aβ_42_-derived diffusible ligands (ADDLs) were prepared by incubating Aβ_42_ for 24 h, as previously described.^25^ For the circular dichroism (CD) analysis, the samples containing ADDLs were dialyzed overnight against 20 mM sodium phosphate buffer, 150 mM NaCl, pH 7.4, 4 °C, with 3 kDa MWCO membranes.

### Dynamic Light Scattering (DLS)

WT-TTR, M-TTR, and Aβ_40_ samples were prepared at a final protein concentration of 15 μM in 20 mM phosphate buffer, 150 mM NaCl, pH 7.4, 25 °C. Before the measurements, the protein samples were filtered with 20 nm cutoff Anotop filters (Whatman, Little Chalfont, U.K.). DLS measurements were performed using a Zetasizer Nano S device from Malvern Instruments (Malvern, Worcestershire, U.K.) thermostated with a Peltier system. Low-volume 10 × 4 mm^2^ disposable cells were used, and the values of refractive index and viscosity set on the instrument were determined using the software provided with the instrument, based on the information of buffer and temperature provided by the user. All size distributions were the average of three consecutive measurements.

### Time Course of Aβ_40_ Amyloid Fibril Formation

Solutions of monomeric Aβ_40_ were prepared by dissolving the lyophilized peptide in 6 M guanidinium hydrochloride (GdnHCl). Monomeric forms were purified from potential oligomeric species and salt using a Superdex 75 10/300 GL column (GE Healthcare) at a flow rate of 0.5 mL min^–1^ and were eluted in 20 mM phosphate buffer, 150 mM NaCl, pH 7.4. The center of the peak was collected, and the peptide concentration was determined from the absorbance of the integrated peak area using ε_280_ = 1490 M^–1^ cm^–1^.

The Aβ_40_ samples were diluted to final concentrations of 1–5 μM with 20 mM phosphate buffer, 150 mM NaCl, pH 7.4, at 37 °C, under quiescent conditions, and supplemented with a small volume from a 2 mM thioflavin T (ThT) stock solution in water to reach a final ThT concentration of 20 μM. WT-TTR and M-TTR were added to final monomer equivalents (m.e.) ranging from 0.01 to 0.5 relative to Aβ_40_. All samples were prepared in low-binding Eppendorf tubes on ice using careful pipetting to avoid introduction of air bubbles. Each sample was then pipetted into multiple wells of a 96-well half-area, low-binding, clear bottom, and poly(ethylene glycol) (PEG)-coated plate (Corning 3881) to give 80 μL per well (three repeats per sample). The 96-well plate was placed at 37 °C under quiescent conditions in a plate reader (Fluostar Omega, Fluostar Optima, or Fluostar Galaxy, BMG Labtech, Offenburg, Germany). The ThT fluorescence was measured in real time for all of the wells concomitantly through the bottom of the plate with a 440 nm excitation filter and a 480 nm emission filter. The time course (or kinetic trace, ThT fluorescence versus time) of aggregation was then obtained for each well.

### Chemical Kinetics Model of Aβ_40_ Amyloid Fibril Formation

Amyloid fibril formation was analyzed using a kinetic model containing the microscopic steps of primary nucleation and fibril elongation and a multistep secondary nucleation, the mechanism of aggregation of Aβ_40_, as previously described^26,27^
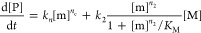
1
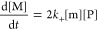
2where [P] and [M] are the number and mass concentrations of aggregates, respectively; *k*_*n*_, *k*_2_, and *k*_+_ are the rate constants for primary nucleation, secondary nucleation, and fibril elongation, respectively; [m] is the concentration of monomers; *K*_M_ is the Michaelis constant; and *n*_c_ and *n*_2_ are the reaction orders relative to the monomer of primary and secondary nucleations, respectively.

All kinetic traces of aggregation obtained at different Aβ_40_ concentrations were first normalized to fibrillar mass concentration, dividing the ThT fluorescence values at time *t* ([M]_*t*_) by the corresponding values measured at the plateau ([M]_∞_), taken as 1.0 or 100%. The resulting traces were analyzed simultaneously with a procedure of global best fitting using the fitting platform amylofit,^26−28^ which employs an integrated rate law obtained from the above equations, as previously described^26−28^
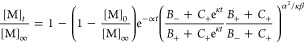
3where the definitions of the parameters are
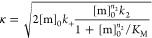
4
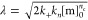
5
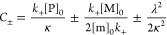
6
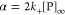
7
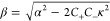
8
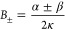
9and where [m]_0_ is the initial monomer concentration; [P]_0_, [M]_0_ and [P]_∞_, [M]_∞_ are the aggregate number and mass concentration at the beginning and end of aggregation, respectively; *k*_+_, *k*_*n*_, and *k*_2_ are the rate constants of elongation, primary, and secondary nucleations, respectively; *n*_c_ and *n*_2_ are the reaction orders relative to the monomer of primary and secondary nucleations, respectively; and *K*_M_ is the Michaelis constant; other details were as reported previously.^27^ The dependence of the aggregation half-time (*t*_1/2_) on [m]_0_ can be expressed as a simple scaling law *t*_1/2_ ∼ ([m]_0_)^γ^, with the scaling exponent γ. For the above model, γ is given by^27^
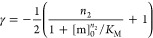
10where γ = −*n*_2_ + 1/2 at low monomer concentrations and γ = −1/2 at high monomer concentrations.^26,27,29^

The global fitting procedure was first performed using the kinetic traces of Aβ_40_ at different concentrations in the absence of the TTR species to obtain the mean values and errors for *K*_M_, *k*_+_*k*_*n*_, and *k*_+_*k*_2_ that best describe all kinetic traces. It was then repeated on the kinetic traces in the presence of 5.0 μM Aβ_40_ and the TTR species at different concentrations to identify the microscopic steps inhibited by each TTR form. This was achieved by allowing one of the microscopic rate constants *k*_*n*_, *k*_2_, or *k*_+_ to vary between data sets recorded at different inhibitor concentrations and forcing the other parameters to be global, i.e., take the same value for all data sets, as outlined in detail.^28^ It was then repeated on the kinetic traces in the presence of 5.0 μM Aβ_40_, 25% Aβ_40_ seeds, and TTRs at different concentrations to assess whether the TTR species affected the fibril elongation process. It was finally repeated on the kinetic traces in the presence of 5.0 μM Aβ_40_, 2% Aβ_40_ seeds, and TTRs at different concentrations to assess whether the TTR species affected the secondary nucleation process. This was achieved by comparing the *k*_2_ values required to describe the aggregation kinetics, in both the absence and presence of the TTR species. Finally, to assess the consistency of our conclusions from both the unseeded and lightly seeded experiments, the data at 5.0 μM Aβ_40_ in both the absence and presence of 2% Aβ_40_ seeds, and in the absence and presence of 0.1 m.e. of either M-TTR or WT-TTR, were fitted together. The rate constants *k*_*n*_ and *k*_2_ were both allowed to vary (while *k*_+_ was kept constant) but forced to take the same value for both the unseeded and the seeded data.

### Atomic Force Microscopy (AFM) Imaging

High-resolution and phase-controlled AFM was performed on positively functionalized mica (TedPella Inc.) substrates.^30^ The mica surface was cleaved and incubated for 1 min with 10 μL of 0.5% (v/v) (3-aminopropyl)triethoxysilane (APTES) from Sigma-Aldrich (St. Louis, MO) in Milli-Q water. Then, the substrate was rinsed three times with 1 mL of Milli-Q water and dried by a gentle stream of nitrogen gas. Aliquots of an Aβ_40_ sample aggregating at a concentration of 10 μM in 20 mM sodium phosphate buffer, 150 mM NaCl, pH 7.4, 37 °C, with or without 0.08 m.e. of M-TTR were removed from the aggregation reaction at 0, 1, 3, and 4 h of incubation and were directly deposited onto the functionalized mica surfaces. The droplets were incubated for 10 min, then rinsed with 1 mL of Milli-Q water, and dried by the gentle stream of nitrogen gas. The preparation was carried out at room temperature. AFM maps were realized by means of a JPK nanowizard2 (Berlin, Germany) system operating in tapping mode and equipped with a silicon tip (μmasch, 2 N m^–1^) with a nominal radius of 10 nm. Image flattening was performed by SPIP (Image Metrology, Hørsholm, Denmark) software.

### Labeling of TTRs with *N*-(7-Dimethylamino-4-methylcoumarin-3-yl)maleimide (DACM)

Each TTR variant was diluted to 0.2 mM in 20 mM phosphate buffer, 50 mM NaCl, pH 7.4, 25 °C. Aliquots of DACM dissolved in pure DMSO were added to a 10-fold molar excess of dye. Each sample was wrapped with aluminum foil and incubated under shaking for 1 h at 37 °C. The reaction was quenched with 5 μL of trifluoroacetic acid (TFA). The unbound dye was removed by extensive dialysis, using 3 kDa MCWO membranes, and the sample was then centrifuged to remove any precipitate. The DACM concentration of the resulting labeled protein sample was determined using ε_381_ = 27 000 M^–1^ cm^–1^. The protein concentration was measured at 280 nm using the same ε_280_ values reported above, after subtraction of the contribution of an equimolar concentration of DACM-GSH. Only samples with a degree of labeling close to 100% were used. The absence of free DACM, unlabeled protein, and multiply labeled protein in the samples, as well as the presence of singly labeled TTRs, was checked with mass spectrometry, as previously reported.^31^

### Intrinsic Fluorescence and Fluorescence Resonance Energy Transfer (FRET)

Aliquots of an Aβ_40_ sample aggregating at a concentration of 10 μM in 20 mM sodium phosphate buffer, 150 mM NaCl, pH 7.4, 37 °C, were removed from the various aggregation reactions at different time points and mixed with solutions containing either DACM-labeled or unlabeled WT-TTR, M-TTR, or W79F-M-TTR. The final conditions after mixing were 9 μM Aβ_40_ and 3 μM (0.3 m.e.) DACM-labeled or unlabeled TTR monomer (in one of its three forms), at molar ratios of 1:3 (TTR/Aβ_40_) in 20 mM sodium phosphate buffer, 150 mM NaCl, pH 7.4, 37 °C. Fluorescence emission spectra (excitation 290 nm) were recorded using a PerkinElmer LS 55 spectrofluorimeter (Waltham, MA) equipped with a thermostated cell holder attached to a Haake F8 water bath (Karlsruhe, Germany). A 10 × 2 mm^2^ quartz cuvette was used. The FRET efficiency (*E*) and the associated distance (*R*) between Cys10-DACM and Trp41 were determined as determined previously.^31^

In another set of experiments, Aβ_42_-derived diffusible ligands (ADDLs) were prepared by incubating Aβ_42_ for 24 h as described.^25^ 12 μM Aβ_42_ ADDLs (m.e.) were co-incubated with 4 μM DACM-labeled or unlabeled M-TTR or W79F-M-TTR (m.e.) at molar ratios of 1:3 (TTR/Aβ_42_) in 20 mM sodium phosphate buffer, 150 mM NaCl, pH 7.4, 37 °C. 4 μM DACM-labeled or unlabeled M-TTR or W79F-M-TTR (m.e.) was also incubated under the same conditions in the absence of Aβ_42_ ADDLs. 12 μM Aβ_42_ ADDLs (m.e.) were also incubated in the absence of the TTR species. Fluorescence emission spectra were recorded at 0, 20, 40, and 60 min and analyzed using the same apparatus, cuvette, and equation as described above. Proteolytic activity of M-TTR on monomeric Aβ_42_ was excluded by incubating the two proteins for 30 min or 4 h before SDS-PAGE analysis.

### Far-UV CD Spectroscopy

Far-UV CD spectra of WT-TTR, M-TTR, and W79F-M-TTR in the presence of Aβ_40_ following a 30 min co-incubation in 20 mM sodium phosphate buffer, pH 7.4, 25 °C, and of all four proteins incubated for 30 min individually, were collected from 185 to 250 nm using a J-810 spectropolarimeter from Jasco (Tokyo, Japan) equipped with a thermostated cell holder attached to a Thermo Haake C25P water bath (Karlsruhe, Germany) and using a 0.1 cm path length cuvette (Hellma, Müllheim, Germany). NaCl (150 mM) was not used in this case due to the noise generated by this salt in the far-UV CD spectra. Total protein concentrations of 0.1 mg mL^–1^ were used for both individual proteins or protein mixtures (TTR-Aβ_40_); these corresponded to 23 and 7.3 μM (individual proteins) or 11.2 and 3.7 μM (protein mixtures at molar ratios of 3:1) for Aβ_40_ and TTR monomers, respectively. All spectra were blank-subtracted and converted to mean residue ellipticity ([Θ]). In the case of protein mixtures, mean residue ellipticity values [Θ] were calculated as
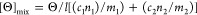
11where Θ is the ellipticity in mdeg units, *l* is the path length in cm units and has a value of 0.1 cm, *n*_1_ and *n*_2_ are the numbers of residues of TTR and Aβ_40_, respectively, *m*_1_ and *m*_2_ are the molecular masses in Da of TTR and Aβ_40_, respectively, and *c*_1_ and *c*_2_ are the protein concentrations in mg mL^–1^ of TTR and Aβ_40_, respectively. The theoretical average mean residue ellipticity values ([Θ]_avg_), assuming that neither unstructured to structured transitions nor secondary structure rearrangements occur, were calculated as
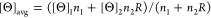
12where [Θ]_1_ and [Θ]_2_ correspond to the measured mean residue ellipticity values, *n*_1_ and *n*_2_ to the number of residues of TTR and Aβ_40_, respectively, and *R* to the excess molar ratio of protein.

In another set of experiments, far-UV CD spectra of 4 μM M-TTR and W79F-M-TTR incubated in the presence of 12 μM (m.e.) Aβ_42_ ADDLs in 20 mM sodium phosphate buffer, pH 7.4, 37 °C, were recorded after 0, 20, 40, and 60 min using a J-810 spectropolarimeter from Jasco (Tokyo, Japan) equipped with a thermostated cell holder attached to a Thermo Haake C25P water bath (Karlsruhe, Germany) and using a 0.1 cm path length cuvette (Hellma, Müllheim, Germany). All spectra were blank-subtracted and converted to mean residue ellipticity ([Θ]).

### Fluorescence Quenching of M -TTR by Aβ_42_ ADDLs

Fluorescence spectra of M-TTR (excitation 280 nm, emission 300–450 nm) at an initial concentration of 7.2 μM were recorded after adding increasing volumes of 20 mM phosphate buffer, 150 mM NaCl at 37 °C, with or without Aβ_42_ ADDLs, with final concentrations of the latter ranging from 0 to 9 μM (m.e.). A 10 × 4 mm^2^ quartz cuvette was used with the same fluorimeter apparatus as that described above. We also acquired fluorescence spectra of ADDLs alone to confirm the absence of fluorescence. The ratio between the total emitted fluorescence (300–450 nm) in the absence (*F*_0_) and presence (*F*) of ADDLs was determined at all ADDL concentrations.

### Size Exclusion Chromatography

Aβ_42_ ADDLs (1.0 mg mL^–1^) and M-TTR (0.2 mg mL^–1^) were mixed or kept separated and incubated at 25 °C for 30 min in PBS. Five hundred microliters were then injected into a Superdex 200 Increase 10/300 GL (Ge Healthcare, Little Chalfont, U.K.) and run with an AKTA-pure 25 L (GE Healthcare), with PBS elution and optical absorption recording at 280 nm. One milliliter fractions were collected, precipitated with prechilled acetone, and analyzed by SDS-PAGE with a 4–20% Mini-PROTEAN precast protein gel (Bio-Rad, Hercules, CA). The obtained gel was stained with silver nitrate.

### Cell Toxicity Assays

The 3-(4,5-dimethylthiazol-2-yl)-2,5-diphenyltetrazolium bromide (MTT) reduction assay was performed as described previously.^13,32^ The image acquisition and analysis of caspase-3 activity were also performed as described previously.^13,32^

## Results

### Aβ_40_, WT-TTR, and M-TTR Are Not Aggregated After Purification

Samples of Aβ_40_, WT-TTR, and M-TTR were generated as described in Materials and Methods, and analysis with SDS-PAGE showed that the proteins were electrophoretically pure. An initial preliminary analysis was carried out with dynamic light scattering (DLS) to rule out the possibility that any of the three purified proteins had aggregated prior to the analysis. The Aβ_40_ peptide was found to have an apparent hydrodynamic diameter (*D*_h_) of 3.0 ± 0.1 nm (Supporting Information, Figure S1), in agreement with previously reported values^33^ and with the expectations originating from general polymer scaling arguments for a monomeric unfolded 40-residue long peptide, i.e., 3.5 ± 0.7^34^ or 3.6 ± 1.7 nm.^35^ M-TTR was found to have a *D*_h_ value of 4.2 ± 0.1 nm (Supporting Information, Figure S1), in good agreement with values previously determined experimentally^22,36,37^ and with the value theoretically expected for a monomeric 127-residue globular protein, i.e., 3.9 ± 0.9 nm.^35^ WT-TTR was found to have a *D*_h_ value of 6.2 ± 0.1 nm (Supporting Information, Figure S1), which is in good agreement with that expected for the folded tetrameric state, as calculated from geometric laws.

### Secondary Nucleation Step in Aβ_40_ Aggregation Is Saturated Under the Used Conditions

**Figure 1:**
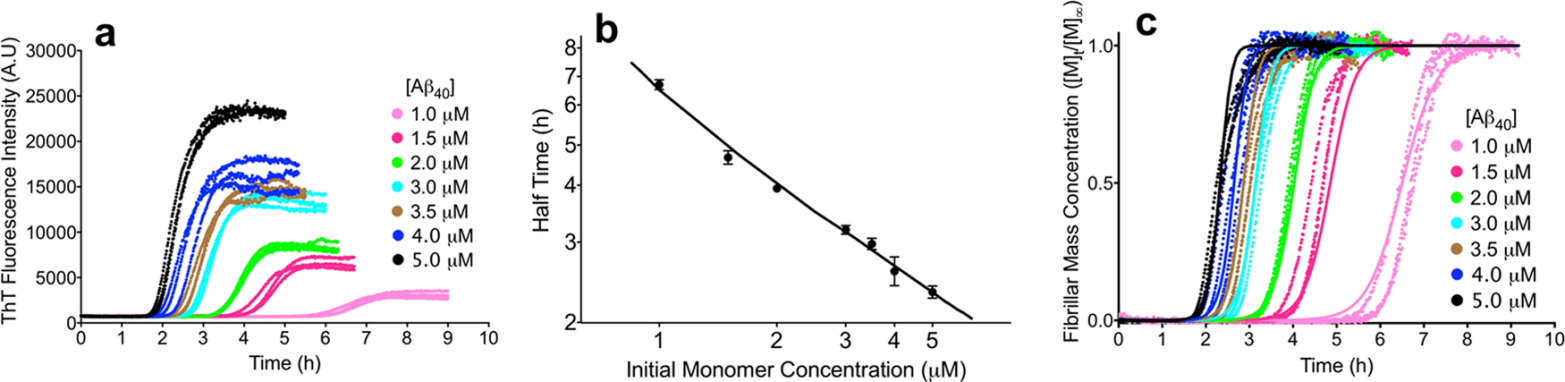
Secondary nucleation step in Aβ_40_ aggregation is kinetically saturated. (a) Time courses of amyloid fibril formation at the indicated Aβ_40_ concentrations, (b) half-time of Aβ_40_ aggregation versus the initial monomer concentration, as derived from (a). The solid line is the theoretical prediction of the half-time using eq 10. Error bars are standard deviations (s.d.). (c) Normalized time courses as derived from (a). The solid lines show predictions for the resulting reaction profiles using a kinetic model containing the microscopic steps of primary nucleation and fibril elongation and a multistep secondary nucleation (eq 3) with fixed reaction orders of *n*_c_ = *n*_2_ = 2.^27^

We then monitored the aggregation of Aβ_40_ at various concentrations in a buffer close to physiological conditions and probed the progress of the aggregation reaction using ThT fluorescence at 480 nm (excitation 440 nm). Specifically, Aβ_40_ was incubated at a series of concentrations between 1.0 and 5.0 μM in 20 mM phosphate buffer, 150 mM NaCl, 20 μM ThT, pH 7.4, at 37 °C, under quiescent conditions. The ThT fluorescence values of the resulting samples were measured at regular time intervals and plotted as a function of time (Figure 1a). In previous work, ThT was not found to significantly affect the rate of fibril formation of Aβ_40_.^27^ In all cases, the time courses followed a characteristic sigmoidal profile with lag, exponential, and plateau phases. The length of the lag phase was found to decrease with the Aβ_40_ concentration, and the steepness of the exponential phase increased, as well as the final ThT fluorescence measured at the plateau, which is proportional to the peptide concentration to a good approximation (Figure 1a).^38^ Three samples were prepared for each Aβ_40_ concentration, and the resulting kinetic traces were found to show little variation (Figure 1a).

To interpret quantitatively these time courses, we considered first the half-time of the fibril mass formation as a function of the initial monomer concentration, which yields the exponent γ that informs on the dominant nucleation mechanisms active in a given system.^27^ In the case of Aβ_40_ aggregation, it has been found to vary from −1.2 at low Aβ_40_ concentrations to −0.2 at high Aβ_40_ concentrations.^27^ This is due to the fact that at low monomer concentrations, where γ is highly negative, rates of secondary nucleation are predominantly determined by the attachment of free monomers to the surface of the fibrils, and thus the rates depend strongly on the monomer concentration. Instead, at high monomer concentrations, where γ is lower, secondary nucleation is saturated, as secondary nucleation sites are fully occupied by monomers. Under these conditions, the rate of nucleation is no longer dependent on the concentration of free monomers but is governed by the rate of conversion and detachment of the bound monomers on the fibril surface.^27^ Interestingly, the data in Figure 1b show an exponent γ above −1.0 (less negative), at all monomer concentrations, indicating that secondary nucleation is likely to have approached saturation under the conditions explored here. This saturation at lower concentrations than those observed previously is likely a result of the increased ionic strength under the conditions used here. As established previously, an increase in ionic strength promotes the attachment step during the secondary nucleation of Aβ and thus leads to saturation at lower concentrations.^39^

We next proceeded to assess if all kinetic traces could be quantitatively reproduced from a molecular model in which saturated secondary nucleation is the mechanism dominating the kinetics of aggregation, as suggested by the scaling analysis discussed above. To this effect, all of the kinetic traces were first normalized to the same fibrillar mass concentration by dividing the ThT fluorescence intensity at time *t* ([M]_*t*_) by the corresponding values measured at the plateau ([M]_∞_), taken as 1.0 or 100% (Figure 1c). Oligomer concentrations were found to be small compared to monomer and fibrillar concentrations and thus do not need to be considered explicitly in this normalization.^40^ The resulting time courses of [M]*_t_*/[M]_∞_ at different Aβ_40_ concentrations were then analyzed simultaneously by generating the best fits to the experimental data using a single rate law (eq 3) that was derived from a model that includes the specific microscopic steps of primary nucleation, fibril elongation, and a saturated secondary nucleation process (eqs 1 and 2), as described previously^27^ and in Materials and Methods (Figure 1c). From the fitting of the model, we also found that the concentration at which secondary nucleation has reached half-saturation (*K*_m_^1/2^) was below the range of Aβ_40_ concentrations studied here (1–5 μM), in agreement with the idea that the saturation of the secondary nucleation pathway is expected at the measured monomer concentrations.

### WT-TTR and M-TTR Inhibit Primary Nucleation in the Process of Aβ_40_ Aggregation

**Figure 2:**
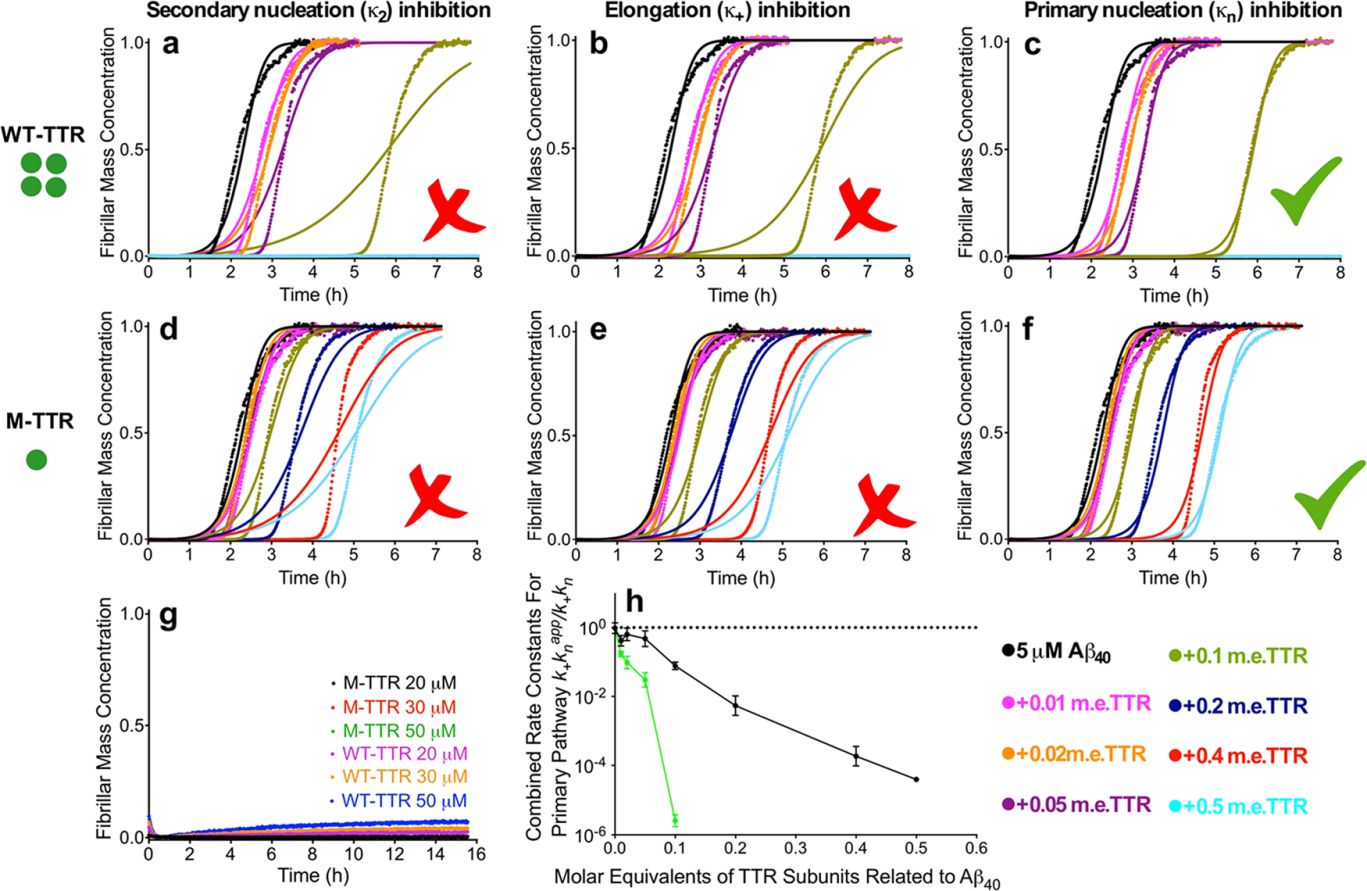
WT-TTR and M-TTR display strong inhibition of primary nucleation in Aβ_40_ aggregation. (a–f) Time courses of fibril formation by 5.0 μM Aβ_40_ in the presence of WT-TTR (a–c) and M-TTR (d–f) at the indicated concentrations (m.e.). The solid lines are fits of the kinetic profiles by a model in which secondary nucleation (a, d), elongation (b, e), or primary nucleation (c, f) is inhibited by TTRs. (g) Incubation of 20, 30, and 50 μM WT-TTR and M-TTR under the same conditions in the absence of Aβ_40_. (h) Change in the effective rate constants of primary nucleation in Aβ_40_ aggregation, as derived from (e) and (f), shown with increasing concentrations of WT-TTR (green) and M-TTR (black). Error bars are s.d. of the rates obtained from individual fitting of the repeats. The traces in (a)–(f) show the average traces of those repeats.

The time courses of amyloid fibril formation by 5.0 μM Aβ_40_ were studied under the same solution conditions in the absence and presence of tetrameric WT-TTR and monomeric M-TTR at different concentrations, ranging from 0.01 to 0.5 molar equivalents (m.e.) of monomeric TTR subunits (Figure 2a–f). All concentrations of TTR molecules reported here refer to concentrations of monomeric TTR subunits. The fibril formation kinetics of a 5.0 μM sample of Aβ_40_ were found to be significantly slowed down in the presence of either TTR variant, but the comparison of kinetic traces at similar m.e. shows that WT-TTR has a more effective inhibitory effect. When the two forms of TTR studied here were incubated under the same experimental conditions in the absence of Aβ_40_, the ThT fluorescence was not found to increase with time, even with very high concentrations of M-TTR and WT-TTR (Figure 2g).

We then carried out a quantitative analysis of the effects of the two different TTR species by fitting the rate law determined in the absence of TTR but allowing some of the rates to vary (see Materials and Methods).^26,41^ The kinetic aggregation traces in Figure 2a–f could be fitted to the moment equation after introducing suitable perturbations to single rate constants describing individual microscopic processes (eq 3). This approach thereby indicated which specific microscopic steps were affected by the presence of the TTR species and showed that the unseeded aggregation kinetics of Aβ_40_ in the presence of a range of M-TTR or WT-TTR concentrations investigated here are very well described when the primary nucleation rate constant (*k*_*n*_) is specifically decreased (Figure 2c,f). By contrast, the experimental data are not consistent with predictions made by altering the rate constants of secondary nucleation (*k*_2_) or elongation (*k*_+_) (Figure 2a,b,d,e).

This analysis reveals that in the presence of M-TTR or WT-TTR, the primary nucleation pathway, which is governed by the product of the rate constants of elongation and primary nucleation (*k*_+_*k*_*n*_), is specifically perturbed. In particular, the *k*_+_*k*_*n*_ value in the presence of the TTR species (*k*_+_*k*_*n*_^app^) was found to decrease by a factor of approximately 10 and 10^6^ with 0.1 m.e. of WT-TTR and M-TTR, respectively, relative to that in the absence of TTRs (*k*_+_*k*_*n*_) (Figure 2h and Supporting Information, Table S1). Hence, the analysis indicates that the inhibitory effects of WT-TTR and M-TTR on unseeded Aβ_40_ aggregation originate largely from the modulation of a single key microscopic step, i.e., primary nucleation.

### WT-TTR and M-TTR Do Not Modify the Elongation Step in Aβ_40_ Aggregation

**Figure 3:**
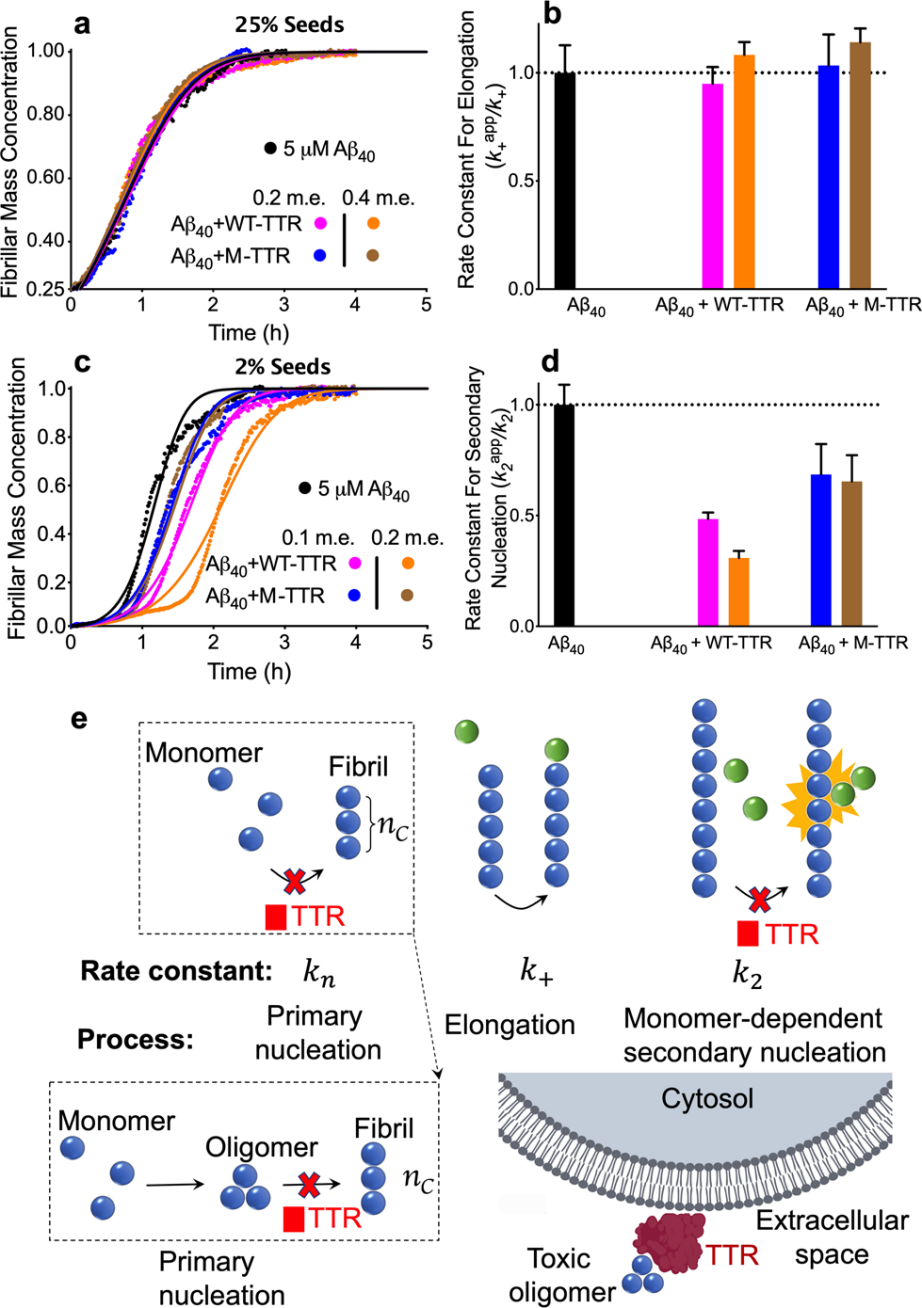
WT-TTR and M-TTR cause significant inhibition of secondary nucleation, but not elongation, in Aβ_40_ aggregation. (a) Kinetic profiles of a 5.0 μM Aβ_40_ solution with 25% of preformed seeds (m.e.) in the absence and presence of 0.2 or 0.4 m.e. of M-TTR and WT-TTR. Solid lines are fits of the reaction profiles when elongation (*k*_+_) is allowed to vary by the different TTR species. (b) Normalized rate constants for fibril elongation derived from the fitted curves in (a). Error bars are s.d. (c) Kinetic profiles of a 5.0 μM Aβ_40_ solution with 2% of preformed seeds (m.e.) in the absence and presence of 0.1 or 0.2 m.e. of M-TTR or WT-TTR. Solid lines represent predictions for the resulting reaction profiles when secondary nucleation (*k*_2_) is allowed to vary by the different TTR species. (d) Normalized rate constants for secondary nucleation derived from the fitted curves in (c). Error bars are s.d. (e) Scheme of the proposed protective mechanisms operated by WT-TTR and M-TTR on Aβ fibril formation and its toxicity. Top: microscopic processes of amyloid fibril formation and associated rate constants slowed down by WT-TTR and M-TTR (red crosses). Bottom right: binding of TTR molecules to Aβ oligomers and inhibition of their toxicity.

The analysis described so far identified primary nucleation as the most strongly affected kinetic quantity. To probe whether there was also a small effect on elongation rate (*k*_+_) that was not visible in the unseeded data due to the strong effect on primary nucleation, we recorded time courses of Aβ_40_ fibril formation under the same conditions, but in the presence of large quantities of preformed fibrils of Aβ_40_, corresponding to 25% of soluble Aβ_40_ in m.e. and in the presence of 0.2 and 0.4 m.e. of WT-TTR and M-TTR (Figure 3a). With 25% of preformed fibrils, the primary and secondary nucleation steps are effectively bypassed, the overall number of aggregates remains approximately constant in time, and the dominant mechanism responsible for the consumption of monomeric Aβ_40_ is the elongation of the preformed fibrils.^40,42^ In the absence of TTR, amyloid fibril formation by Aβ_40_ was very rapid and without a detectable lag phase, as expected due to the presence of seed fibrils (Figure 3a). Neither 0.2 nor 0.4 m.e. of WT-TTR and M-TTR was found to affect significantly the aggregation kinetics (Figure 3a,b), revealing that Aβ_40_ fibril elongation is not affected by the presence of either TTR form.

### WT-TTR and M-TTR Inhibit Secondary Nucleation in Aβ_40_ Aggregation

To probe whether there was also an effect on secondary nucleation rate constant (*k*_2_) in the presence of the two forms of TTR that was not visible in the unseeded data due to the strong effect on primary nucleation, we repeated the experiments described above in the presence of smaller quantities of preformed fibrils of Aβ_40_ (2% m.e.). At this concentration of fibril seeds, primary nucleation is bypassed, but both fibril elongation and surface-catalyzed secondary nucleation contribute significantly to the overall kinetics of fibril formation.^42−45^ In the absence of either form of TTR, the entire process of Aβ_40_ fibril formation was found to have an intermediate rate relative to the corresponding processes in the absence and presence of 25% seeds, with a short lag phase still detectable (Figure 3c). In the presence of 0.1 and 0.2 m.e. of M-TTR and WT-TTR, the fibril formation process was significantly slowed down in a concentration-dependent manner, with the efficiency of inhibition being higher for WT-TTR than for M-TTR (Figure 3c,d). Since neither M-TTR nor WT-TTR affects Aβ_40_ fibril elongation at these concentrations (Figure 3a,b), and the primary nucleation step is bypassed with 2% fibrillar seeds, this retardation can be attributed to a decrease in the rate constant of secondary nucleation (*k*_2_). Indeed, the experimental time courses are well described when only *k*_2_ is varied in the presence of increasing concentrations of M-TTR and WT-TTR (Figure 3c,d). The *k*_2_ values were found to be decreased by ca. 32 and 35% at 0.1 and 0.2 m.e. of M-TTR, respectively, and by ca. 52 and 70% at 0.1 and 0.2 m.e. of WT-TTR, respectively (Figure 3d). As this is a small decrease compared to the decrease of primary nucleation, it was not observed in unseeded experiments, where the inhibition of primary nucleation dominates. As a further check, we show how the unseeded and the 2% seeded traces can be described by the simultaneous inhibition of both primary and secondary nucleations (Supporting Information, Figure S2). These fits confirm the inhibition of both primary and secondary nucleations.

In conclusion, the agreement between the experimental traces and their fits suggests that the inhibition of Aβ_40_ fibril formation observed in the unseeded experiments by WT-TTR and M-TTR is largely due to an inhibition of primary nucleation and, in part, secondary nucleation (Figure 3e). The efficiency of this inhibition was found to be different for the two forms of TTR, however, as the comparison of the kinetic plots obtained at the same concentrations of TTR molecules in terms of monomeric subunits shows that WT-TTR is able to inhibit both primary and secondary nucleations to a greater extent than M-TTR.

### M-TTR Delays the Deposition of Amyloid Fibrils of Aβ_40_

**Figure 4:**
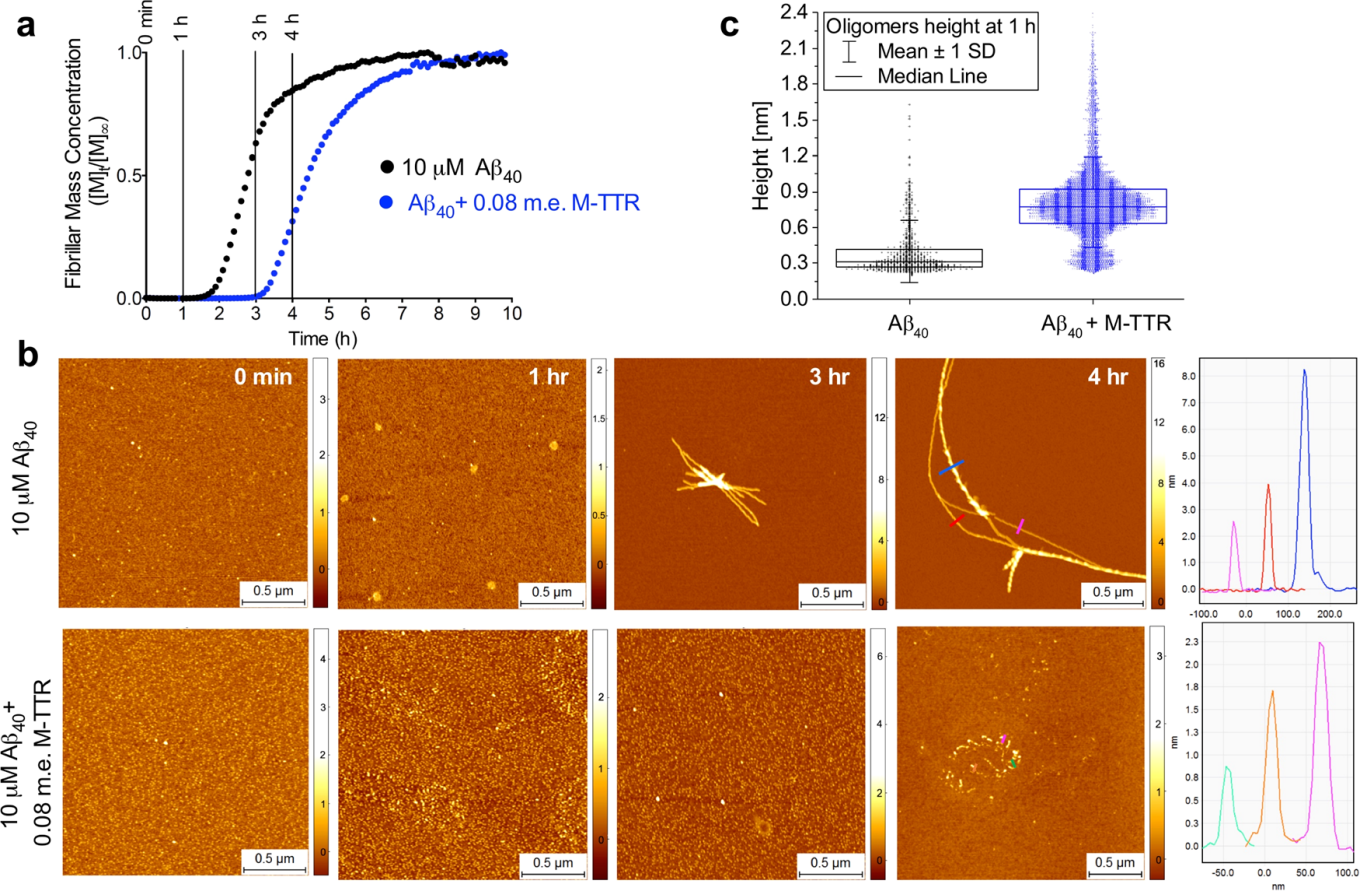
M-TTR delays the formation of Aβ_40_ amyloid fibrils. (a) Time course of ThT fluorescence for 10 μM Aβ_40_ with and without 0.08 m.e. of M-TTR. The vertical bars indicate the time points of sample withdrawal for AFM imaging. (b) AFM images at 0, 1, 3, and 4 h of aggregation after a twofold dilution. (c) Statistical analysis and box-chart representation of the height of the oligomers after 1 h, as evaluated from AFM imaging.

The aggregation process of Aβ_40_ was also probed using phase-controlled and high-resolution atomic force microscopy (AFM), with and without M-TTR to follow the time-dependent appearance of the various aggregate morphologies. Aβ_40_ was incubated under the same solution conditions at a concentration of 10 μM in the absence or presence of 0.08 m.e. of M-TTR, and samples were withdrawn and analyzed at various times between 0 and 4 h (Figure 4a). We did not perform the same experiment with WT-TTR because the aggregation process was still in the lag phase under these conditions after 4 h (Figure 2a). In the absence of M-TTR, after incubation for 1 h, corresponding to the lag phase in the ThT fluorescence time course, aggregated species with a height of 0.2–0.4 nm were the predominant species, attributable to the individual polypeptide chains in a monomeric state deposited on the mica surface; however, a small but significant population of species with a height of 0.5–1.0 nm was present and can be attributed to oligomers (Figure 4b,c). Indeed, such species are likely to have a larger diameter in suspension than that estimated here, as a consequence of both the minor deformations caused by the cantilever tip on the oligomer shape and of the discoidal shape of the oligomers that results from the fact that AFM measures accurately their height rather than their width. After incubation for 3 h, i.e., during the exponential phase in the ThT fluorescence time course, a modest population of species with a height of 2–4 nm, attributable to protofibrils and protofilaments, is clearly present. After incubation for 4 h, corresponding to the plateau phase, a large population of mature fibrils, with a height of approximately 6–9 nm, dominates the sample, along with some of the protofilaments with a height of 2–4 nm^46−48^ (Figure 4b).

In the presence of 0.08 m.e. of M-TTR, amyloid fibril formation was substantially slower than in its absence, and monomers with a height of 0.2–0.4 nm persisted after 3 h and were still the predominant species after 4 h, where only a small number of short, 2–3 nm high protofibrils were detected (Figure 4b). Nevertheless, larger numbers of oligomers were observed during the lag phase in the presence of M-TTR, as after 1 h of incubation species with heights of 0.5–1.2 nm were predominant, and even larger oligomers with heights greater than 1.2 nm were detected (Figure 4b,c and Supporting Information, Figure S3). These results indicate that M-TTR inhibits the process of amyloid fibril formation by Aβ_40_.

### WT-TTR and M-TTR Undergo a Specific Conformational Change Upon Binding to Aβ_40_ Oligomers

We next studied the intrinsic fluorescence of TTRs during Aβ_40_ fibril formation to probe possible conformational changes of TTRs when interacting with Aβ_40_. WT-TTR and M-TTR have two tryptophan (Trp) residues in each monomer subunit, at positions 41 and 79, whereas Aβ_40_ does not have any, making it possible to attribute the observed intrinsic fluorescence entirely to TTR molecules. Moreover, only Trp41 is fluorescent in the native state because Trp79 fluorescence is naturally quenched.^31,49,50^ For this reason, we analyzed a W79F mutant of M-TTR, which contains the only fluorescent Trp41, avoiding complications arising from Trp79 fluorescence increases during conformational changes.

**Figure 5:**
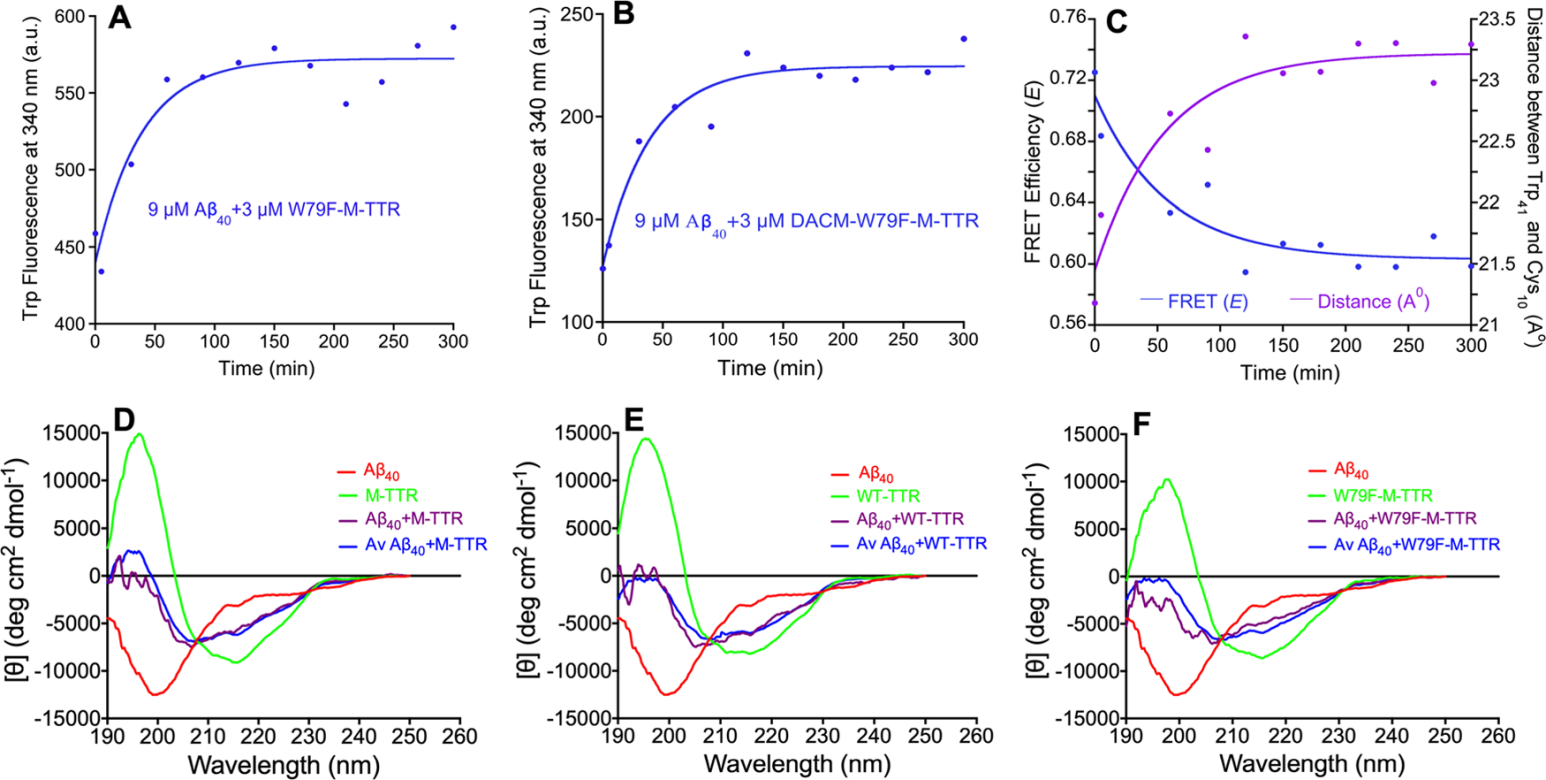
TTRs undergo a conformational change, but maintain their topology, upon binding to Aβ_40_ oligomers. (A, B) Time course of Trp intrinsic fluorescence measured after mixing an aliquot of Aβ_40_ undergoing aggregation (at the indicated time points) and an aliquot of an unlabeled (A) or DACM-labeled (B) W79F-M-TTR. The solid lines result from a procedure of best fitting using single-exponential functions. (C) Time courses of FRET *E* (blue) and Cys10-Trp41 distance *R* (purple) using the values derived from (A) and (B). The solid lines are best-fits to single-exponential functions. (D–F) Far-UV CD spectra measured after incubating Aβ_40_ with WT-TTR (D), M-TTR (E), and W79F-M-TTR (F), for 30 min using a total protein concentration of 0.1 mg mL^–1^ and using a 1:3 molar ratio of TTR/Aβ_40_ (purple). Spectra of Aβ_40_ alone (red) or TTR alone (green) are also shown. Spectra reconstructed from a linear combination of the spectra of Aβ_40_ alone and TTR alone using a 1:3 molar ratio, as described in Materials and Methods, are also shown (blue).

Aβ_40_ (10 μM) was incubated under the same conditions as described above. At regular time intervals, aliquots were withdrawn and mixed with W79F-M-TTR. The final conditions were 9 μM Aβ_40_, 3 μM (0.3 m.e.) W79F-M-TTR, 150 mM NaCl, pH 7.4, 25 °C. The Trp intrinsic fluorescence was then measured immediately after mixing and plotted versus time of Aβ_40_ aggregation (Figure 5A). As Aβ_40_ aggregation proceeds, Trp fluorescence increases until an apparent equilibrium is reached. This occurs with an apparent rate constant of 4.3(±1.3) × 10^–4^ s^–1^, indicating that such a change occurs within the lag phase of Aβ_40_ fibril formation. Since M-TTR does not bind monomeric Aβ_40_,^7^ the observed change can be attributed to its binding to Aβ_40_ in an oligomeric state.

The analysis was then repeated under identical experimental conditions using W79F-M-TTR covalently labeled with *N*-(7-dimethylamino-4-methylcoumarin-3-yl)maleimide (DACM) at Cys10 (Figure 5B). DACM acts as an acceptor of the fluorescence emitted from Trp41 due to FRET.^31^ The initial Trp fluorescence at time 0, in the absence of Aβ_40_, is very low due to FRET (compare the *y* scales in Figure 5A,B). The Trp fluorescence then increases with a rate similar to that of the unlabeled mutant, i.e., 4.3(±1.0) × 10^–4^ s^–1^. By determining the FRET efficiency (*E*) values at any given time using the Trp fluorescence values reported in Figure 5A,B and by converting such values into distance *R* between Cys10-DACM and Trp41, plots of *E* vs time and *R* vs time could be reconstructed,^31^ showing a time-dependent decrease of the FRET *E* value attributable to a conformational change that increases slightly, but significantly, the distance between Cys10 and Trp41 (Figure 5C).

### Overall Fold of WT-TTR and M-TTR Is Unchanged During the Lag Phase of Aβ_40_ Aggregation

We next used far-UV circular dichroism (CD) to probe possible changes in the secondary structure of the various forms of TTR when interacting with Aβ_40_. In particular, we acquired far-UV CD spectra of the three forms of TTR alone and after co-incubation for 30 min with Aβ_40_, a time sufficient for the TTR molecules to interact with oligomeric Aβ_40_, as indicated by the intrinsic fluorescence measurements (Figure 5D–F). The total protein concentration was 0.1 mg mL^–1^, corresponding to 23 and 7.3 μM for Aβ_40_ and TTR monomers, respectively, in 20 mM phosphate buffer, pH 7.4, 25 °C. The far-UV CD spectra after incubating WT-TTR and Aβ_40_ for 30 min are largely superimposable with that obtained by averaging the spectra recorded for the two proteins individually (Figure 5D). This is in agreement with a previous report on a similar time scale.^8^ Similar conclusions were obtained when the analysis was repeated with M-TTR (Figure 5E) and W79F-M-TTR (Figure 5F). Hence, the interaction of all three forms of TTR with Aβ_40_ during the lag phase of its aggregation process does not even partially disrupt the secondary structure of the TTR species.

### Different Forms of TTR Maintain Their Topology and Secondary Structure Upon Binding to Toxic Aβ_42_ Oligomers

Previous studies have shown that WT-TTR and M-TTR can interact with preformed toxic oligomers of Aβ_40_ and Aβ_42_ and inhibit their toxicity.^7,12,13^ We therefore repeated our biophysical analysis using preformed amyloid-derived diffusible ligands (ADDLs) formed by Aβ_42_ that have previously been shown to be toxic to rat neuronal cells, rat hippocampal neurons, and mice organotypic hippocampal slice cultures.^12,25,51−53^ The identity of our ADDL preparations was confirmed by SDS-PAGE and Western blotting (Supporting Information, Figure S4a), as previously reported.^51^ The TTR molecules that we used were M-TTR and its mutant W79F-M-TTR because M-TTR was found to be the most effective form for suppressing the toxicity of Aβ_42_ oligomers.^13^ The binding of M-TTR with the Aβ_42_ ADDLs was checked with two independent tests (Supporting Information, Figure S4b–e).

**Figure 6:**
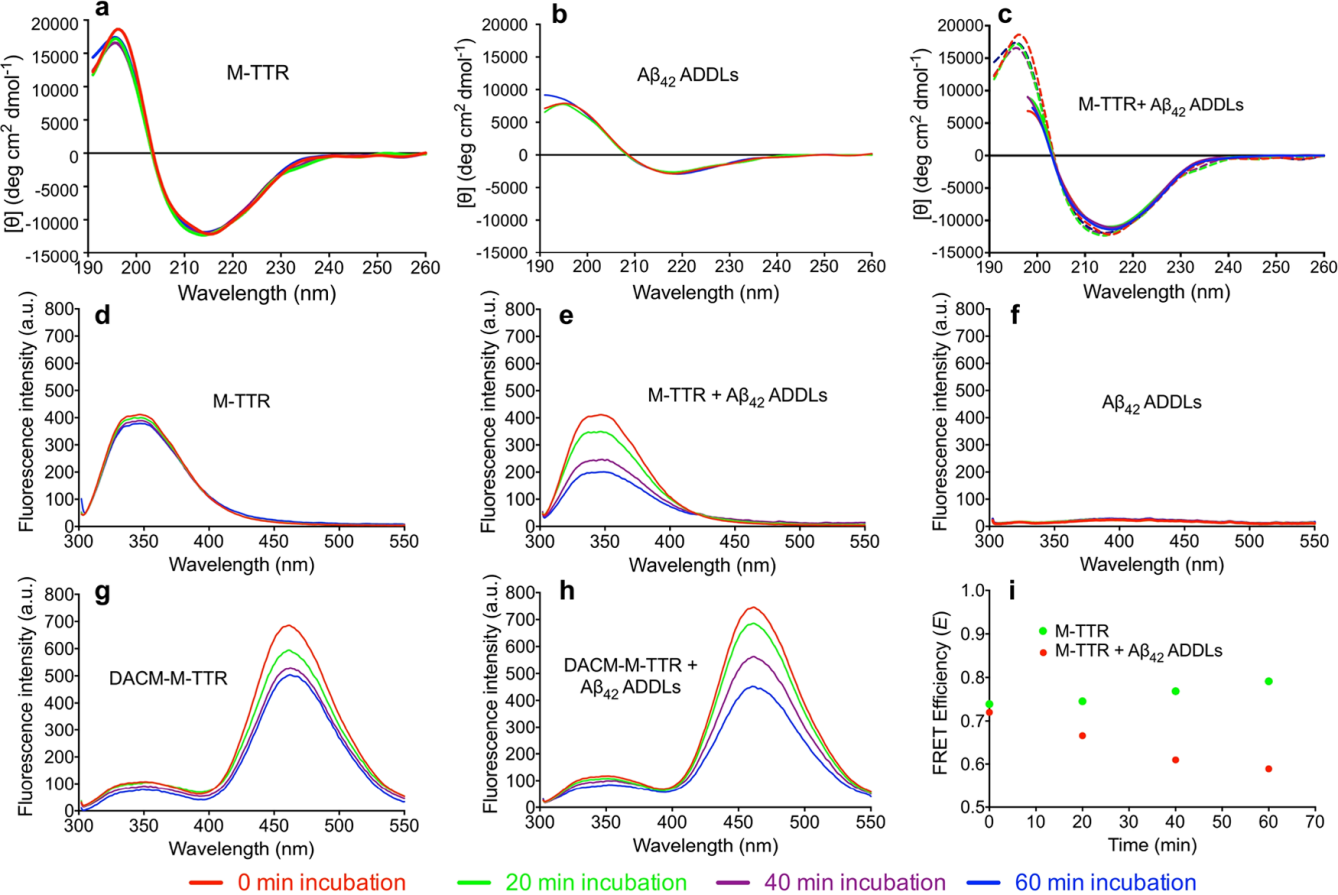
M-TTR undergoes a specific conformational change, while maintaining the topology, upon binding to Aβ_42_ ADDLs. (a–c) Far-UV CD spectra of M-TTR (a), Aβ_42_ ADDLs (b), and M-TTR plus Aβ_42_ ADDLs (c) measured at the indicated times of incubation using a protein concentration of 0.1 mg mL^–1^ (a, b) or 0.2 mg mL^–1^ with a 1:1 mass ratio of M-TTR: Aβ_42_ ADDLs (c). In panel (c), the experimental spectra (continuous lines) are compared with those obtained by summing the spectra of M-TTR alone and ADDLs alone (dashed lines). (d–h) Fluorescence spectra recorded for both M-TTR (d, e) and DACM-M-TTR (g, h) in the absence (d, g) and presence (e, h) of Aβ_42_ ADDLs after the indicated times of incubation. Fluorescence spectra recorded for Aβ_42_ ADDLs alone are also shown (f). (i) FRET *E* values of M-TTR in the absence (green) and presence (red) of Aβ_42_ ADDLs.

M-TTR (4 μM) was then incubated with or without preformed Aβ_42_ ADDLs (12 μM, m.e.) in 20 mM phosphate buffer, 150 mM NaCl, pH 7.4, at 37 °C, and far-UV CD spectra were recorded after 0, 20, 40, and 60 min. The spectrum of M-TTR was found to be stable with time and to be typical of an all-β protein, as described above (Figure 6a). The spectrum of the Aβ_42_ ADDLs in the absence of M-TTR was also stable with time and had a low value of mean residue ellipticity with a negative peak at 218 nm, indicating a significant level of organized secondary structure (Figure 6b). The spectrum of Aβ_42_ ADDLs in the presence of M-TTR was stable with time and similar to that obtained by adding together the spectra of M-TTR alone and ADDLs alone (Figure 6c). The analysis was repeated for W79F-M-TTR, leading to very similar results (Supporting Information, Figure S5a–c), confirming that no change of secondary structure and topology occurs for M-TTR or W79F-M-TTR following their interaction with Aβ_42_ ADDLs.

### Various TTR Species Undergo a Specific Conformational Change Upon Binding to Toxic Aβ_42_ Oligomers

The interaction between ADDLs and M-TTR was also studied by means of intrinsic fluorescence and FRET using the same experimental conditions as those described in the previous section. The fluorescence spectra of both M-TTR and DACM-M-TTR were found to be stable within the first hour of incubation in the absence of the ADDLs (Figure 6d,g), whereas those recorded in their presence decreased in intensity with time (Figure 6e,h), indicating a progressive interaction of M-TTR and DACM-M-TTR with the ADDLs. The ADDLs were not fluorescent, indicating that this species does not contribute to the observed fluorescence spectra (Figure 6f). The FRET *E* value of M-TTR in the absence of the ADDLs was stable with time (Figure 6i) and similar to that measured previously,^31^ whereas that in their presence decreased progressively (Figure 6i). This analysis was repeated for the W79F mutant of M-TTR, leading to very similar results (Supporting Information, Figure S5d–i). These results indicate that a conformational change occurs for M-TTR or its mutant following the interaction with ADDLs and also indicate an increased spatial distance between DACM-Cys10 and the two tryptophan side chains, especially Trp41.

### Various TTR Species Suppress the Toxicity of Aβ_42_ Oligomers

We then evaluated whether the interaction between TTR and ADDLs was able to suppress the toxicity of the oligomers. We first added preformed ADDLs to the cell culture medium of human SH-SY5Y neuroblastoma cells at a concentration of 12 μM (m.e.), confirming their toxicity using the 3-(4,5-dimethylthiazol-2-yl)-2,5-diphenyltetrazolium bromide (MTT) assay (Supporting Information, Figure S6a). We then treated the cells with ADDLs preincubated for 1 h in the presence of 1.2 μM WT-TTR or M-TTR (m.e.) and found no detectable toxicity, with a level of MTT reduction similar to that of untreated cells or to cells treated with the Aβ_42_ monomers (Supporting Information, Figure S6a). Similar results were obtained from analyzing caspase-3 activity, a well-recognized apoptotic marker, using confocal microscopy (Supporting Information, Figure S6b). Fluorescence microscopy images and the corresponding semiquantitative values of the green fluorescence signal associated with caspase-3 activity show that WT-TTR and M-TTR significantly prevented the apoptotic response induced by ADDLs (Supporting Information, Figure S6b). These results confirm the ability of M-TTR and WT-TTR to suppress the toxicity of ADDLs (Figure 3e), as previously reported.^13^

## Discussion

### TTR Inhibits Primary and Secondary Nucleations of Aβ by Binding to Oligomers and Preventing Their Conversion into Short Fibrils

In this work, we have confirmed the inhibition of the process of amyloid fibril formation by Aβ_40_ that is mediated by monomeric and tetrameric forms of TTR,^6,7,10^ but have, in addition, studied this process further by investigating the microscopic mechanisms underlying this phenomenon. We have shown that this inhibitory effect results from the ability of the different forms of TTR to slow down the microscopic steps of both primary and secondary nucleations while leaving the elongation step unaffected (Figure 3e).

How can we interpret these results? Under the conditions of highly substoichiometric concentrations of WT-TTR and M-TTR used here, nearly all of the Aβ_40_ molecules will be unbound in solution, regardless of the binding affinity. The lack of any effect of WT-TTR and M-TTR on the rate of the microscopic step of fibril elongation observed here and previously^54^ is consistent with the hypothesis that binding between TTR molecules and Aβ_40_ monomers, if present, is not significant in determining the kinetics of Aβ_40_ fibril formation. Hence, the ability of WT-TTR or M-TTR to inhibit primary nucleation cannot be attributed to their ability to sequester Aβ_40_ monomers from the formation of nuclei competent for growth and elongation. Both WT-TTR and M-TTR can, however, bind Aβ_40_ oligomeric species with high affinity,^5−8^ suggesting that both forms of TTR can bind to the Aβ_40_ oligomeric species formed in the aggregation process, preventing their structural reorganization into species that are able to elongate (Figure 3e). Moreover, this conclusion is consistent with (i) the observation made in this study with AFM that M-TTR can stabilize oligomeric Aβ_40_, (ii) with the recent finding that M-TTR can bind to oligomers and block their conversion into fibrils,^9^ and (iii) with the previous finding that WT-TTR can bind to oligomers that are positive for the OC antibody and block their growth, maturation, or reorganization;^8^ indeed, such OC-positive oligomers have been shown to be off-pathway species, thus unproductive for fibril elongation.^55^ Indeed, such off-pathway intermediates would be expected to dissociate more slowly than the overall time scale of the aggregation reaction, and further studies are needed to address the metastability of the TTR-bound oligomers. Overall, blockage of primary nucleation does not arise in this case from monomer sequestration but by interaction with oligomeric species and inhibition of their conversion and growth into nuclei.

The lack of effect on Aβ_40_ fibril elongation indicates that TTR molecules do not bind strongly to the growing ends of Aβ_40_ fibrils. By contrast, a significant effect on secondary nucleation was observed under conditions of saturation of the Aβ_40_ binding sites on fibril surfaces, in agreement again with the concept that the various forms of TTR hinder the conversion of fibril-bound Aβ_40_ oligomers into species able to elongate. Different mechanisms of action have been proposed for TTR, as well as extracellular chaperones, in the binding of protein-misfolded oligomers and inhibition of their toxicity, including “binding followed by further oligomer clustering” and “binding causing hydrophobic shielding”.^56^ Both of these mechanisms have indeed been observed for M-TTR and toxic HypF-N or Aβ_42_ oligomers at particular protein:protein molar ratios.^8,9,13,57^ The AFM results reported here indicate that M-TTR can stabilize the smaller Aβ_40_ oligomers, as well as induce the formation of larger Aβ_40_ assemblies, consistent with these two views.

In our conditions of analysis, M-TTR was found to be less effective than WT-TTR in inhibiting Aβ_40_ fibril formation, in apparent disagreement with an earlier report under similar conditions.^7^ The difference can be due to subtle differences in experimental conditions, as well as in differences in TTR and Aβ_40_ protein purification.

### M-TTR Undergoes a Subtle Conformational Change When Interacting with Aβ Oligomers

M-TTR has been shown to be very plastic, with conformational changes occurring as the pH decreases to values of 3.9–5.0,^58^ upon the addition of small amounts of urea at equilibrium before the major unfolding transition at pH 7.4^37^ and transiently during folding from the fully unfolded to the fully folded state at pH 7.4.^37^ The data presented here suggest that M-TTR undergoes a subtle conformational change when interacting with Aβ_40_ and Aβ_42_ oligomers. Far-UV CD indicates that M-TTR maintains its secondary structure and overall topology, but fluorescence measurements reveal that it undergoes subtle changes in conformation, as indicated by small enhancements in tryptophan fluorescence and increases in the average spatial distances between the tryptophan residues, particularly Trp41, and the fluorophore attached to Cys10. Thus, following interaction with Aβ_40_ oligomers forming during amyloid fibril formation and stable Aβ_42_ ADDLs, the FRET efficiency decreases from 0.72 ± 0.02 to 0.60 ± 0.03 and 0.57 ± 0.03, respectively, a change that is less dramatic than that observed upon full unfolding of M-TTR for the same donor–acceptor pair, where the change is from 0.72 ± 0.02 to 0.29 ± 0.01.^31^

This conformational state is distinct from other partially folded or native-like states observed previously. In fact, the amyloidogenic state of M-TTR at pH 3.9–5.0 has the same secondary structure and Trp-DACM FRET efficiency as the native state.^31^ The conformational state populated in 1 M urea at pH 7.4 has a far-UV CD spectrum distinct from that of the native structure, indicating some degree of unfolding, but indistinguishable fluorescence properties, including similar FRET *E* values.^31,37^ Finally, the kinetic folding intermediate at pH 7.4 has a higher FRET efficiency than, and a similar β-sheet content to, the native state, again showing a distinction from the species characterized here.^31,37^ Such a variety of partially folded states adopted by M-TTR under the various circumstances emphasizes the structural plasticity of this protein.

### How Does TTR Prevent Aβ Fibril Formation and Toxicity in Vivo?

The concentration of WT-TTR is estimated to be 0.09–0.36 μM in the human CSF of healthy individuals.^59^ WT-TTR is also present in the neurons and brain parenchyma, although its concentration in these environments has not yet been determined.^11^ By contrast, the concentration of Aβ_40_ in the CSF of healthy individuals is 0.6–5.0 nM^60^ and has been determined to be in the high picomolar to low nanomolar range in the human brain,^5,61,62^ where the concentration of Aβ_42_ is even lower.^60^ The interaction between WT-TTR and monomeric Aβ_40_ occurring in solution has been studied previously^7,10^ and reported to have a *K*_D_ value of 435 ± 19 μM, using Trp41 intrinsic fluorescence quenching by Aβ_40_,^10^ or 24 μM, using solution NMR and isothermal titration calorimetry.^7^ With these protein concentrations and affinities, therefore, only a small fraction of monomeric Aβ_40_ is likely to be associated with tetrameric WT-TTR, i.e., ca. 0.4–1.5% in the CSF, assuming a *K*_D_ value of 24 μM.^7^ A similar order of magnitude (or even lower) is expected in the brain parenchyma, and the situation for monomeric Aβ_42_ is likely to be very similar. Although quantitatively negligible, however, it cannot be excluded that the binding of monomeric Aβ_40_/Aβ_42_ to tetrameric TTR can become significant in some circumstances.^7^

The most effective mechanism inhibiting amyloid fibril formation by Aβ_40_ and Aβ_42_ in vivo is likely to be the interaction between WT-TTR (or its dissociated subunits) and Aβ_40_ or Aβ_42_ oligomers. It is still unclear whether WT-TTR binds the Aβ oligomers in its tetrameric form^7^ or only after dissociation into its constituent subunits.^8^ The affinity of WT-TTR and M-TTR for oligomeric Aβ is much higher than that of monomers.^5−9^ Consequently, such binding can be an effective strategy through which TTR inhibits both Aβ fibril formation (by blocking the conversion of small-sized oligomers into small fibrils through primary and secondary nucleations) and reducing the toxicity of the oligomers (Figure 3e). This latter mechanism of neuroprotection mediated by TTR has been widely described previously^7,11−13^ and has been confirmed in the present study. The former mechanism is as important as the latter, however, because it is increasingly recognized that the proliferation of protein aggregates can be dominated by secondary nucleation on the surfaces of fibrils^42,45^ and that the spread of protein deposition diseases through the brain may result from the diffusion of fibrils.^63,64^

## Conclusions

TTR is increasingly recognized to act as an Aβ scavenger in the brain, as indicated by the increased levels of this protein in the CSF during aging,^16^ the expression of TTR in the heat shock response in neurons,^21^ the concomitant reduction in the level of Aβ and TTR in the CSF in AD,^16,18^ and the colocalization of the two proteins in the senile plaques of the cortex and hippocampus in AD.^11^ In fact, it seems that TTR is upregulated as a result of aging, stress, and as a means of protection against the aggregates of Aβ_40_/Aβ_42_ in the brain and that it follows the fate of the CSF pool of Aβ. In this study, we have shown that an important mechanism of this protective role lies in the ability of TTR to bind to Aβ oligomers and inhibit their conversion into the short fibrils, as both the microscopic steps of primary and secondary nucleations are retarded. This protection, which has been observed to require a subtle conformational conversion of TTR, is dual as it limits both the toxicity of the oligomeric species and the ability of the aggregates to proliferate over time.

## Supporting Information Available

Size distributions of WT-TTR, M-TTR, and Aβ_40_; global fits of the inhibition of TTRs in unseeded and 2% seeded experiments; height of oligomeric populations as measured by AFM; characterization of the identity and M-TTR binding of Aβ_42_ ADDLs; conformational change of W79F-M-TTR upon binding to Aβ_42_ ADDLs; and cell toxicity assays and other experimental details (PDF)

## Abbreviations Used

Aβ: amyloid β
AD: Alzheimer’s disease
ADDLs: amyloid-derived diffusible ligands
AFM: atomic force microscopy
APP: amyloid β precursor protein
CD: circular dichroism
APTES: (3-aminopropyl)triethoxysilane
CSF: cerebrospinal fluid
DACM: *N*-(7-dimethylamino-4-methylcoumarin-3-yl)maleimide
DLS: dynamic light scattering
DMSO: dimethyl sulfoxide
FRET: fluorescence resonance energy transfer
GdnHCl: guanidinium hydrochloride
GSH: glutathione
IPTG: isopropyl β-d-thiogalactoside
m.e.: monomer equivalents
MTT: 3-(4,5-dimethylthiazol-2-yl)-2,5-diphenyltetrazolium bromide
M-TTR: monomeric variant of TTR
TFA: trifluoroacetic acid
TTR: transthyretin
