# Supplementary material for: Transthyretin Inhibits Primary and Secondary Nucleations of Amyloid-β Peptide Aggregation and Reduces the Toxicity of Its Oligomers
Source: Biomacromolecules. 2020 Feb 3;21(3):1112–25. doi: 10.1021/acs.biomac.9b01475 (PMC7997117; doi:10.1021/acs.biomac.9b01475)
Supplement: Supplementary file 1 — bm9b01475_si_001.pdf [file bm9b01475_si_001.pdf]

Supplementary information for

# Transthyretin Inhibits Primary and Secondary Nucleations of Amyloid-β Peptide Aggregation and Reduces the Toxicity of Its Oligomers

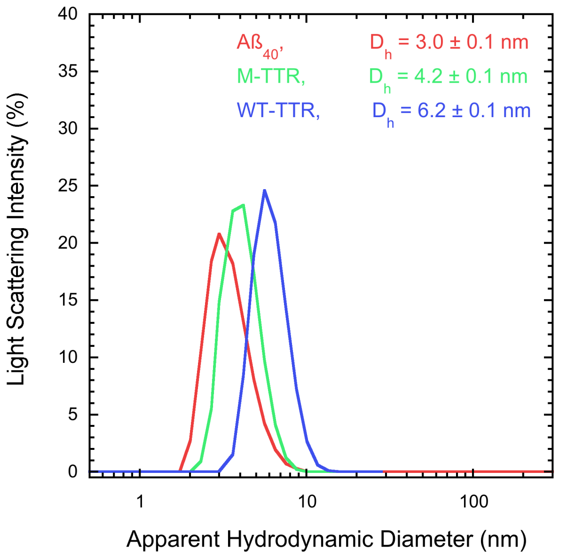


**Fig. S1**. **Size distributions of WT-TTR, M-TTR and A$\beta_{40}$.** Size distributions were obtained with DLS.

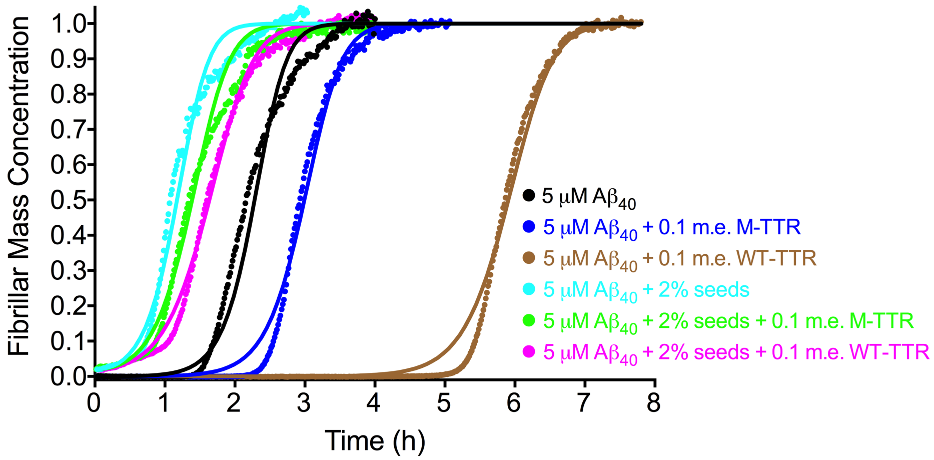


**Fig. S2. Global fits of the inhibition of TTRs in unseeded and 2% seeded experiments.** Kinetic profiles of fibril formation of a 5.0 μM sample of $A\beta_{40}$ in the absence (black) and presence of 0.1 m.e. of WT-TTR (brown) and M-TTR (blue), as well as in the presence of 2% preformed seeds, in the absence (cyan) or presence of 0.1 m.e. of WT-TTR (magenta) or M-TTR (green). Solid lines are fits to the data. These fits were obtained by allowing both the rate of secondary nucleation and the rate of primary nucleation to vary, but forcing them to take the same value for both the seeded and the unseeded data at a given inhibitor concentration. We thus obtain 3 pairs of rate constants; for the uninhibited data, for the data with 0.1 m.e. M-TTR and for the data with 0.1 m.e. WT-TTR. Generally, it is advised to never vary more than one rate when analysing data in the presence of varying inhibitor. However, in this case, the inhibition of both primary and secondary nucleation has been established separately through the use of seeds, thus these feeds here merely constitute a confirmation that all data are indeed consistent with an inhibition of both primary and secondary nucleation.

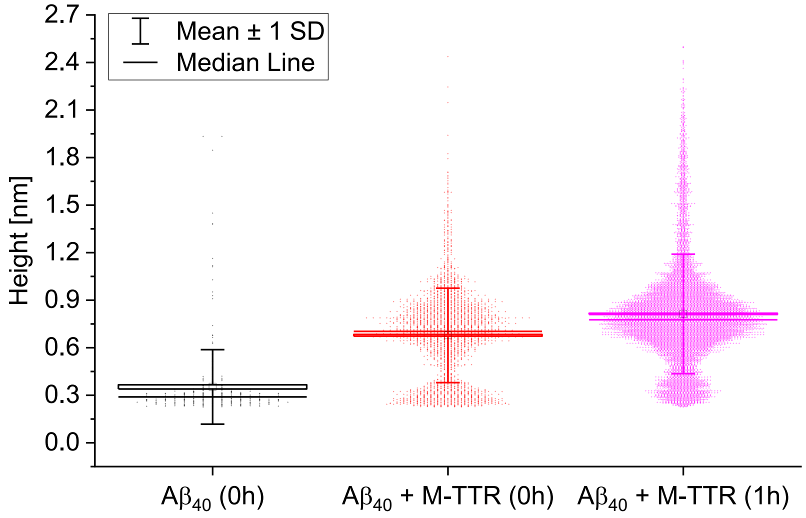


**Fig. S3. Height of oligomeric populations as measured by AFM.** Statistical analysis and box-chart representation of the height of the oligomers of the indicated species and time points, as evaluated from AFM imaging.

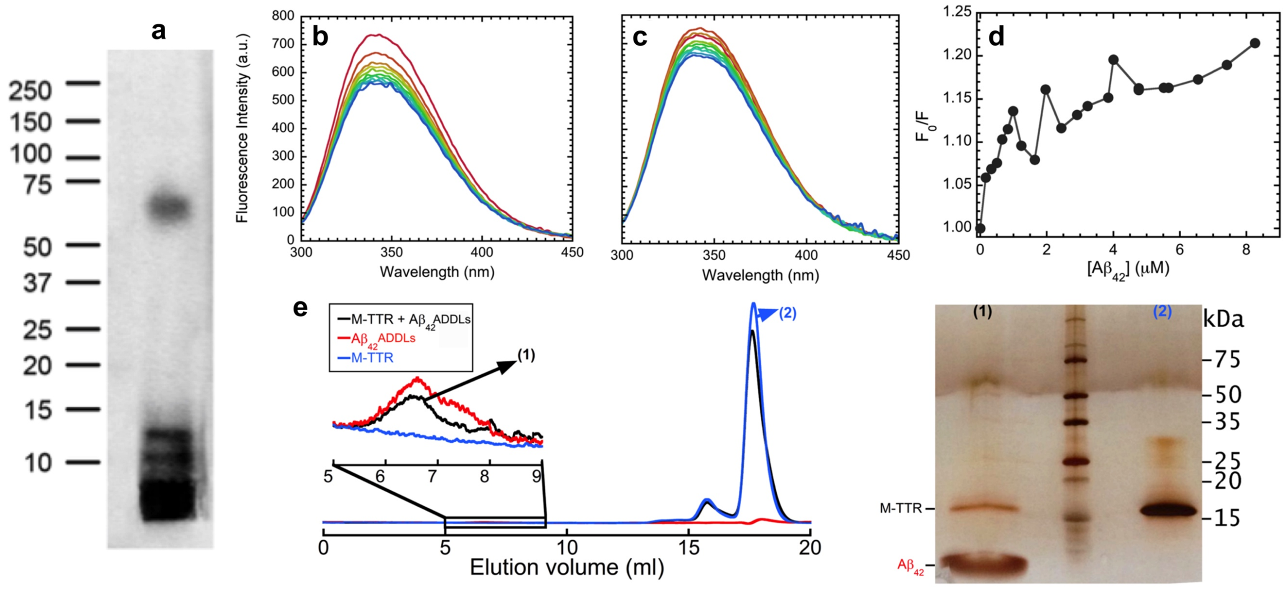


**Fig. S4. Characterisation of the identity and M-TTR binding of $A\beta_{42}$ ADDLs.** (a) Representative Western blotting analysis of $A\beta_{42}$ ADDLs separated by SDS-PAGE on a 4–12% criterion XT Precast Bis–Tris gel and probed with mouse monoclonal 6E10 antibodies and with peroxidase-conjugated anti-mouse secondary antibodies. (b,c) Fluorescence spectra of M-TTR (initial concentration of 7.2 μM) after progressive additions of buffer with (b) or without (c) ADDLs in 20 mM phosphate buffer, 150 mM NaCl at 37 °C, with final concentrations of ADDLs ranging from 0 to 9 μM (m.e.) from red to blue. The excess decrease in fluorescence intensity observed in the presence of ADDLs, relative to that in its absence, is due to the quenching induced by ADDLs on the M-TTR emission intensity, as the latter is only a result of M-TTR dilution. (d) Ratio between the total emitted fluorescence (300-450 nm) in the absence ($F_0$) and presence ($F$) of ADDLs versus ADDLs concentrations (m.e.). If the quenching were simply dynamic the plot would be expected to be linear,[32] which is not the case, indicating that binding of M-TTR to ADDLs occurs even at low concentrations of the latter. (e) Analytical size-exclusion chromatography (SEC) of M-TTR (blue), ADDLs (red) and ADDLs mixed with M-TTR (black). The inset shows the peak corresponding to ADDLs. Fractions containing ADDLs treated with M-TTR (1) and M-TTR alone (2) were run on a SDS-PAGE and stained with silver. Bands corresponding to M-TTR and $A\beta_{42}$ monomer are indicated. M-TTR eluted as a major peak at *ca*. 18 ml with a second minor peak at *ca*. 16 ml, possibly corresponding to a small fraction of dimer or tetramer. The SDS-PAGE profile revealed the presence of M-TTR in the major peak. ADDLs eluted at *ca*. 6-8 ml indicating the presence of oligomers. The mixture of M-TTR and ADDLs also eluted at ca. 6-8 and 18 ml, but the former fraction revealed the presence of both ADDLs and M-TTR in the SDS-PAGE profile, indicating that ADDLs had interacted with a small quantity of M-TTR and had sequestered the protein in the SEC run.

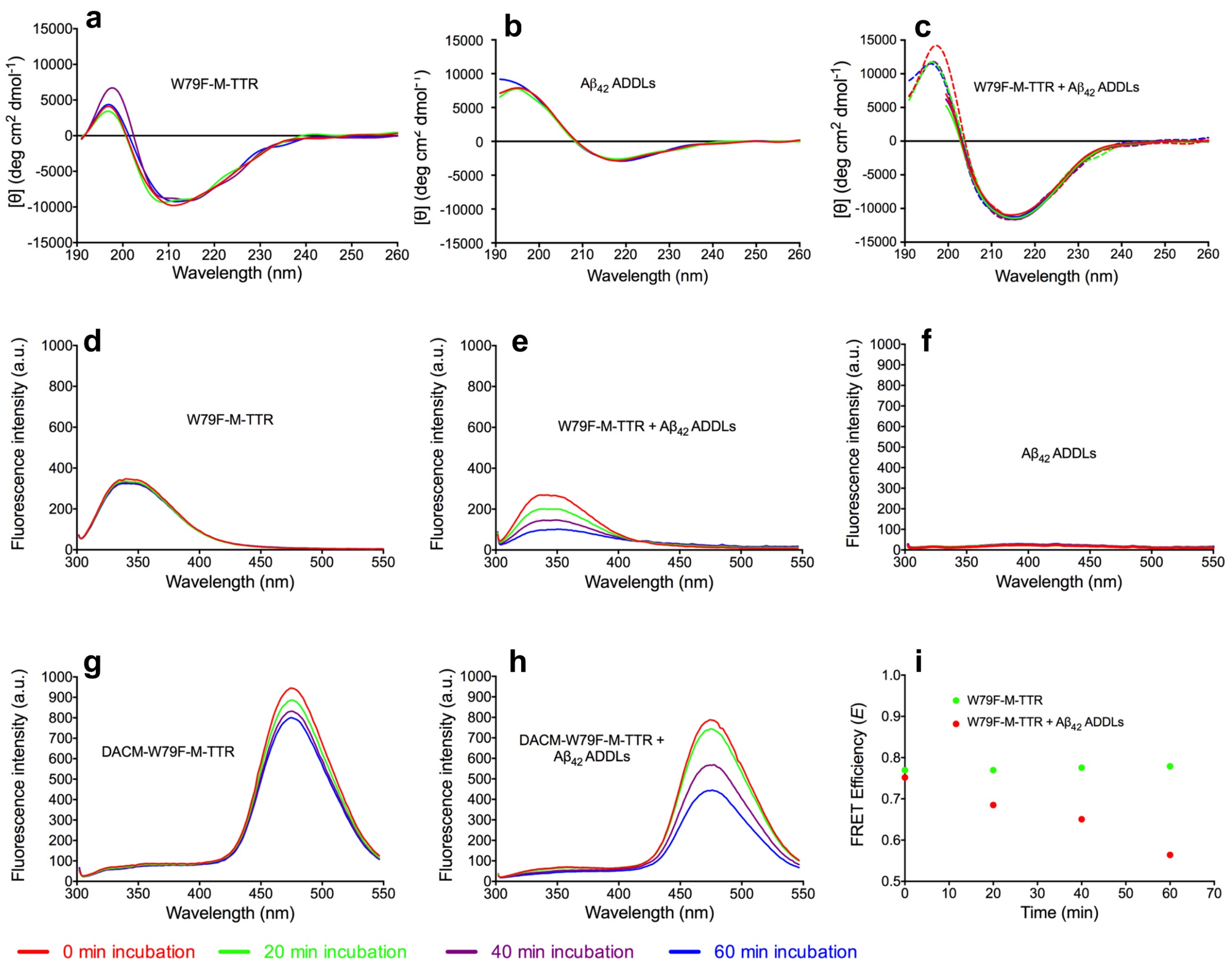


**Fig. S5. W79F-M-TTR undergoes a specific conformational change, while maintaining the topology, upon binding to A$\beta_{42}$ ADDLs.** (a-c) Far-UV CD spectra of W79F-M-TTR (a), A$\beta_{42}$ ADDLs (b) and W79F-M-TTR plus A$\beta_{42}$ ADDLs (c) measured at the indicated times of incubation using a protein concentration of 0.1 mg ml$^{-1}$ (a,b) or 0.2 mg ml$^{-1}$ with 1:1 mass ratio of W79F-M-TTR : A$\beta_{42}$ ADDLs (c). In panel (c) the experimental spectra (continuous lines) are compared with those obtained by adding together the spectra of W79F-M-TTR alone and ADDLs alone (dashed lines). (d-h) Fluorescence spectra recorded for both W79F-M-TTR (d,e) and DACM-W79F-M-TTR (g,h) in the absence (d,g) and presence (e,h) of A$\beta_{42}$ ADDLs after the indicated times of incubation. Fluorescence spectra recorded for A$\beta_{42}$ ADDLs alone are also shown (f). (i) FRET *E* values of W79F-M-TTR in the absence (green) and presence (red) of A$\beta_{42}$ ADDLs.

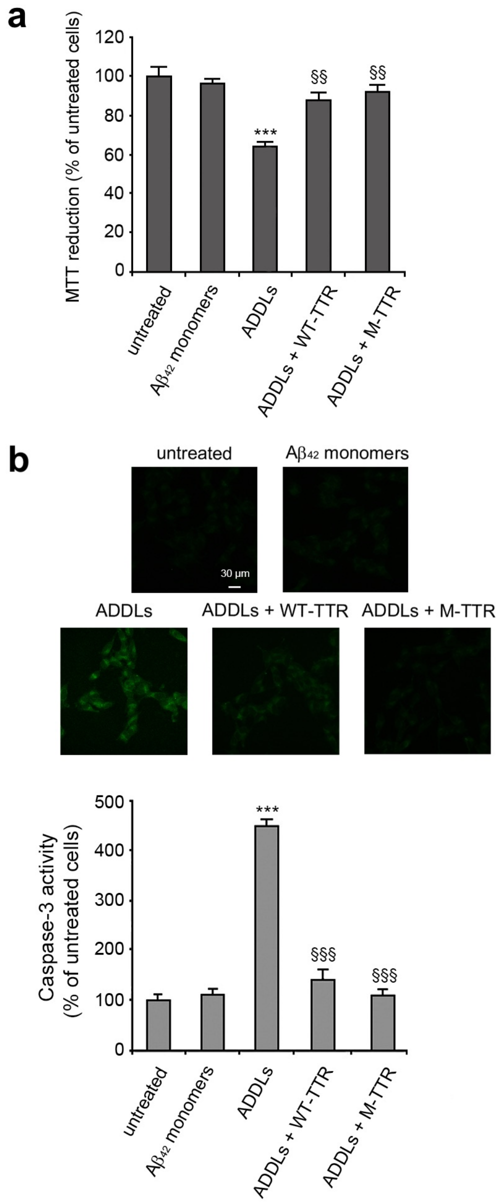


**Fig. S6. Suppression of A$\beta_{42}$ oligomer toxicity by TTRs.** (a) Preformed A$\beta_{42}$ ADDLs were resuspended in the cell culture medium, incubated for 1 h at a corresponding monomer concentration of 12 μM in the absence or presence of 1.2 μM WT-TTR and M-TTR (m.e.) and then added to SH-SY5Y cells for 24 h; the cells were also exposed to A$\beta_{42}$ monomers. Cell viability was expressed as the percentage of the MTT reduction in treated cells compared to untreated cells (taken as 100%). (b) Representative confocal microscope images showing caspase-3 activation in SH-SY5Y cells. Preformed A$\beta_{42}$ ADDLs were resuspended in the cell culture medium, incubated at a corresponding monomer concentration of 12 μM with or without the indicated TTRs (1.2 μM m.e.) for 1 h and then added to SH-SY5Y cells for 24 h. The figure also shows untreated cells and cells exposed for 1 h to A$\beta_{42}$ monomers. Caspase-3 activity (green fluorescence) was assessed using the fluorescent probe FAM-FLICA™ Caspases 3 & 7. The semi-quantitative analysis of the caspase-3-derived fluorescence are expressed as the percentage of the value for untreated cells. Experimental errors are s.d. The *** symbol refers to $p < 0.001$ relative to untreated cells. The symbols §§ and §§§ refer to $p < 0.01$ and $p < 0.001$ relative to cells treated with A$\beta_{42}$ ADDLs.

| Condition | Fitted Value | Condition | Fitted Value |
| --- | --- | --- | --- |
| A$\beta_{40}$ alone | *11 ± 4* $M^{-2}s^{-2}$ | | |
| + 0.01 m.e. M-TTR | *5 ± 2* $M^{-2}s^{-2}$ | + 0.01 m.e. WT-TTR | *1.8 ± 0.3* $M^{-2}s^{-2}$ |
| + 0.02 m.e. M-TTR | *7 ± 3* $M^{-2}s^{-2}$ | + 0.02 m.e. WT-TTR | *1.1 ± 0.5* $M^{-2}s^{-2}$ |
| + 0.05 m.e. M-TTR | *6 ± 3* $M^{-2}s^{-2}$ | + 0.05 m.e. WT-TTR | *0.3 ± 0.2* $M^{-2}s^{-2}$ |
| + 0.1 m.e. M-TTR | *0.8 ± 0.2* $M^{-2}s^{-2}$ | + 0.1 m.e. WT-TTR | $3 \cdot 10^{-5} \pm 1 \cdot 10^{-5}$ $M^{-2}s^{-2}$ |
| + 0.2 m.e. M-TTR | $7 \cdot 10^{-2} \pm 3 \cdot 10^{-2}$ $M^{-2}s^{-2}$ | | |
| + 0.4 m.e. M-TTR | $2 \cdot 10^{-3} \pm 1 \cdot 10^{-3}$ $M^{-2}s^{-2}$ | | |
| + 0.5 m.e. M-TTR | $4 \cdot 10^{-4} \pm 4 \cdot 10^{-5}$ $M^{-2}s^{-2}$ | | |

**Table S1. Combined rate constants for primary pathway of A$\beta_{40}$ fibril formation in the absence and presence of TTR.** Combined rate constants for the primary pathway ($k_+k_n$) were obtained from fitting the experimental curves as obtained in Fig. 2a-f, and errors are expressed as s.d. of the technical replicates. The combined rate constant of secondary pathway ($k_+k_2$) obtained through global fitting for A$\beta_{40}$ both in the absence and presence of TTR was $3.25 \cdot 10^{11}$ $M^{-3}s^{-2}$, and the $\sqrt{K_m}$ value was $5.50 \cdot 10^{-7}$ M.
